# Phenotypic, Transcriptomic, and Metabolomic Signatures of Root-Specifically Overexpressed *OsCKX2* in Rice

**DOI:** 10.3389/fpls.2020.575304

**Published:** 2021-01-20

**Authors:** Huimin Yan, Hongzheng Sun, Xueying Jia, Chuanwei Lv, Junzhou Li, Quanzhi Zhao

**Affiliations:** Henan Key Laboratory of Rice Biology, Collaborative Innovation Center of Henan Grain Crops, Henan Agricultural University, Zhengzhou, China

**Keywords:** *OsCKX2*, cytokinin, transcriptome, metabolome, root-specific expression

## Abstract

Cytokinins are crucial signaling molecules that regulate plant growth and development. *OsCKX2* irreversibly degrades nucleobase cytokinins by encoding cytokinin oxidase/dehydrogenase to control grain production in rice. In this study, *OsCKX2* was specifically overexpressed in roots using *RCc3* promoter to investigate the effects of root-source cytokinins on the growth of rice. *OsCKX2* overexpressed (OE) rice showed retarded growth with lower cytokinin levels and biomass production. Shoot-specific transcriptome analysis between *OsCKX2* OE rice and wild type (WT) revealed differentially expressed genes (DEGs) associated with cell division, cell wall structure, phytohormone signaling, and assimilation and catabolism. Metabolome analysis indicated that a majority of differential primary metabolites, such as amino acids and organic acids, increased, while lipids decreased in *OsCKX2* OE rice. Integration of transcriptomic and metabolomic data showed that several DEGs and differential metabolites were related to glycolysis and tricarboxylic acid cycle (TCA). To conclude, reduced cytokinin levels via root-specific overexpression of *OsCKX2* resulted in developmental defects, which confirmed the importance of root-source cytokinins in plant growth and morphogenesis.

## Introduction

Cytokinins are a class of fundamental phytohormones, which are signaling molecules that modulate various developmental processes. Naturally occurring cytokinins are adenine derivatives and are classified into isoprenoid cytokinins and aromatic cytokinins; the former includes *N6*-(Δ^2^-isopentenyl) adenine (iP), *trans*-zeatin (tZ), *cis*-zeatin (cZ), dihydro-zeatin (DZ) and their riboside, ribotide, or glycoside conjugates. Among these, tZ and iP are ubiquitously and predominantly present in plant species (Hirose et al., 2008). Many studies have illustrated the role of cytokinins and their signaling pathways in almost all the plant growth and developmental processes, such as seed germination (Wang et al., 2011), shoot meristem development (Gordon et al., 2009), root growth (Bielach et al., 2012), chloroplast development (Cortleven and Schmülling, 2015), and reproductive development (Jameson and Song, 2016).

Cytokinin biosynthesis, activation, translocation, and degradation are regulated and maintained by multiple enzymes. Biosynthesis starts with the production of iP-nucleotides by adenosine phosphate-isopentenyltransferases (IPTs), the rate-limiting enzymes (Sakakibara et al., 2005). Cytochrome P450 mono-oxygenase CYP735A hydroxylates and transforms these products into tZ-nucleotides (Hirose et al., 2008). The tRNA isopentenyltransferase (tRNA-IPT) degrades *cis*-hydroxy isopentenyl tRNAs to produce cZ-nucleotides. Nucleoside 5′-monophosphate phosphoribohydrolases, encoded by *LONELY GUY* (*LOG*) genes, catalyze and convert inactive cytokinin nucleotides into active free-base forms in plants and plant-interacting organisms (Kurakawa et al., 2007; Seo and Kim, 2017). Purine permeases (PUPs) and equilibrative nucleoside transporters (ENTs) act as influx carriers to transport cytokinins in an active manner (Liu et al., 2019). Cytokinin oxidase/dehydrogenases (CKXs) preferentially and irreversibly degrade nucleobase cytokinins by cleavage of unsaturated *N6*-isoprenoid side chains to adenines, to control plant cytokinin levels (Ashikari et al., 2005). The multistep His-Asp phosphorelay, which consists of histidine kinase (HK) receptor, histidine phosphotransfer (HP) protein, and separate response regulator (RR), controls the perception and signal transduction of cytokinins (Tsai et al., 2012). Many studies have demonstrated the role of cytokinin signaling genes in plant growth and development. Overexpression of *IPT* in tobacco maintained high water content, retained photosynthetic activity, and retarded leaf senescence under drought conditions (Rivero et al., 2007). Mutation in *CYP735A* genes, involved in *trans*-zeatin biosynthesis in *Arabidopsis thaliana*, resulted in an abnormal lateral root primordia positioning phenotype (Chang et al., 2015). Mutation in LONELY GUY (LOG), a cytokinin-activating enzyme, caused premature termination of shoot meristem and yield reduction in rice (Kurakawa et al., 2007).

In past decades, extensive studies have been carried out on *CKX* gene family, which encodes cytokinin oxidase/dehydrogenase to degrade cytokinins in plants. *CKX2* manipulates endosperm growth to control seed size in *Arabidopsis thaliana* (Li J. et al., 2013). *TaCKX6-D1* and *HvCKX1* play crucial roles in grain weight and yield in wheat and barley, respectively (Zalewski et al., 2010; Zhang et al., 2012). Overexpression of *AtCKX* genes in tobacco and *Arabidopsis* caused remarkable developmental alteration in the shoot and root system (Werner et al., 2001, 2003). Ectopic overexpression of *Arabidopsis thaliana CKX1* elevated drought and heat stress tolerance in tobacco (Macková et al., 2013). In rice, the *CKX* gene family consists of 11 members, and so far only *OsCKX2* and *OsCKX4* have been well-characterized. Studies have demonstrated the role of *OsCKX2* in rice grain production (Ashikari et al., 2005; Li S. Y. et al., 2013). Knockdown of *OsCKX2* decreased grain yield penalty under salinity stress conditions (Joshi et al., 2018). *OsCKX4*, combined with auxin response factor (OsARF25) and cytokinin response regulators (OsRR2 and OsRR3), coordinated crown root formation in rice (Gao et al., 2014).

Cytokinins are mainly biosynthesized in root system, and *OsCKX2* is scarcely expressed in roots (Ashikari et al., 2005; Yeh et al., 2015). It is unknown what the effect of root-specific overexpression of *OsCKX2* on endogenous cytokinin levels and growth and development of rice. Therefore, *OsCKX2* OE rice was constructed using the root-specific promoter *RCc3* (Xu et al., 1995; Gao et al., 2014) to explore the phenotypic changes at seedling and mature stages and to expound the regulatory mechanism of cytokinins through transcriptomics and metabolomics analysis.

## Materials and Methods

### Plant Materials and Growth Conditions

Xinfeng 2 (*Oryza sativa* L. *ssp. japonica*) variety and *OsCKX2* OE rice were used in this study. Wild type (WT; Xinfeng 2) and *OsCKX2* OE rice were grown under greenhouse condition with 14 h, 28°C/10 h, 25°C for light/dark cycle, and natural field condition at Henan Agricultural University research farm, Henan Province, China (34°53′ N, 113°35′ E, 94 m altitude) during the rice-growing seasons with normal crop maintenance practices and rigorous separation measures. Seedlings were cultivated under normal nutrient solution culture condition in the greenhouse to four to five leaf stage, phenotypic data of seedling stage were measured, and plant samples for cytokinin measurement, transcriptome and metabolome analyses were collected, respectively. Phenotypic data at mature stage were measured under natural field condition.

### Plasmid Construction and Rice Transformation

The coding sequence (CDS) of *OsCKX2* (*Os01g0197700*) gene was obtained from the Rice Annotation Project Database (rap-db; http://rapdb.dna.affrc.go.jp/) and optimized and synthesized by Sangon Biotech (Shanghai, China; Supplementary Table 1). The synthetic CDS with *Kpn*I and *Spe*I restriction sites was ligated to pMDC140 vector driven by *RCc3* promoter (Gao et al., 2014) for root-specific expression (Supplementary Figure 1). This plasmid was introduced into *Agrobacterium tumefaciens* strain *EHA105* and subsequently transformed into the scutellar calli of mature Xinfeng 2 seeds to obtain *OsCKX2* OE rice.

### Phenotypic Measurement

Fresh weight, dry weight, grain yield, and 1,000 grain weight were measured using 1/10,000 electronic analytical balance (Sartorius, Beijing, China). Stem thickness was measured using an automatic vernier caliper (SATA, Shanghai, China). Root thickness was evaluated under an Olympus DP27 microscope with CellSense software (Olympus, Tokyo, Japan). Grain length and grain width were measured using a rice appearance quality detector (JMWT12, Dong Fu Jiu Heng, Beijing, China). All phenotypic measurements consisted of 15 biological replicates.

### Cytokinin Measurement

The cytokinin contents were measured using the seedlings at four to five leaf stage and the whole plants at mature stage in WT and *OsCKX2* OE rice. The fresh sample (50 mg) was frozen in liquid nitrogen, ground into powder, and extracted with 0.5 mL methanol/water/formic acid (15:4:1, v/v/v) at 4°C. The extract was vortexed for 10 min and centrifuged at 14,000 rpm for 5 min at 4°C, and the supernatant was collected. The supernatant was further vortexed for 5 min and centrifuged for 5 min at 4°C. Then the extract was evaporated to dryness under nitrogen gas stream, reconstituted in 80% methanol (v/v), ultrasonicated for 1 min, filtrated through PTFE membrane filter (0.22 μm; ANPEL, Shanghai, China) and placed in a sample injector for UPLC-MS/MS analysis using an LC-ESI-MS/MS system (HPLC, Shim-pack UFLC SHIMADZU CBM30A system, http://www.shimadzu.com.cn/; MS, Applied Biosystems 6500 Triple Quadrupole, http://www.appliedbiosystems.com.cn/). The analytical conditions were as follows: HPLC column, Waters ACQUITY UPLC HSS T3 C18 (1.8 μm, 2.1 mm × 100 mm); solvent system, water (added 0.04% acetic acid): acetonitrile (added 0.04% acetic acid); flow rate, 0.35 mL/min; temperature, 40°C; and injection volume, 2 μL. The effluent was connected to an ESI-triple quadrupole-linear ion trap (Q-TRAP)-MS. The ESI source operation parameters were as follows: ion source, turbo spray; source temperature, 500°C; ion spray voltage (IS), 5,500 V; curtain gas (CUR), 35.0 psi; and collision gas (CAD), medium. Three biological replicates were maintained per sample.

### RNA-Sequencing and Statistical Analysis

The shoots of WT and *OsCKX2* OE rice at four to five leaf stage, including the sheaths and leaves, were collected for total RNA extraction with three biological replicates. Total RNA was extracted by Trizol reagent (Invitrogen). Sequence libraries were constructed using NEBNext Ultra RNA Library Prep Kit for Illumina (NEB, USA). The clustering of the index-coded samples was performed using TruSeq PE Cluster Kit v3-cBot-HS (Illumina). The prepared libraries were sequenced on Hiseq X Ten Platform (Illumina). The expression differences between samples were analyzed using DESeq 2.0 (Love et al., 2014). False Discovery Rate (FDR) was obtained by Benjamini-Hochberg method. Genes with |log_2_ fold change| ≥1.0 and false discovery rate (FDR) <0.05 were defined as differentially expressed genes (DEGs) between *OsCKX2* OE rice and WT. Gene Ontology (GO) enrichment analysis on DEGs was done by BiNGO plugin of Cytoscape (Shannon et al., 2003). Each GO term was evaluated by hypergeometric test and Benjamini-Hochberg FDR correction. GO terms with a corrected *P* < 0.01 were considered as significantly enriched. FPKM (fragments per kilobase of transcript per million mapped reads) was deemed an indicator of gene expression levels, and log_2_ (FPKM) values of DEGs were used to draw heatmaps. Pathway analyses of DEGs were conducted according to the Kyoto Encyclopedia of Gene and Genomes (KEGG) (http://www.genome.jp/kegg/) database.

### Gene Expression Analysis

First strand cDNA was synthesized from the extracted RNA using GoScript™ Reverse Transcription System (Promega, Madison, WI) following the manufacturer's instructions. Semi-quantitative PCR (semi-qPCR) was performed using *EasyTaq* DNA Polymerase (TransGen Biotech, Beijing, China) under the following conditions: initial denaturation for 5 min at 95°C; 30 cycles of denaturation for 30 s at 95°C, annealing for 30 s at a temperature dependent on the primers, and elongation for 30 s at 72°C, and final extension for 5 min at 72°C. Subsequently, the expression levels of endogenous and synthetic *OsCKX2* genes were detected with 28 cycles of amplification by agarose gel electrophoresis, and the housekeeping gene, *Actin*, was detected with 30 cycles of amplification in semi-qPCR. Quantitative real-time polymerase chain reaction (qRT-PCR) was carried out on CFX96 Real-Time PCR System (Bio-Rad, Hercules, CA) using GoTaq® qPCR Master Mix (Promega, Madison, WI) according to manufacturer's instructions. All qRT-PCR reactions were repeated three times on three biological replicates, and relative gene expression levels were calculated by 2^−ΔΔCT^ method. Rice *Actin* gene was used as an endogenous control in both semi-qPCR and qRT-PCR. The primers used are listed in Supplementary Table 2.

### Metabolomic Profiling and Statistical Analysis

Freeze-dried shoots of WT and *OsCKX2* OE rice were triturated in a mixer mill (MM 400, Retsch) with a zirconia bead for 1.5 min at 30 Hz. Three biological replicates were analyzed for each sample. The powder (100 mg) was weighed and extracted with 1.0 mL 70% aqueous methanol overnight at 4°C. After centrifugation at 10,000 g for 10 min, the extract was collected using CNWBOND Carbon-GCB SPE Cartridge (ANPEL, Shanghai, China) and filtered through a membrane filter (0.22 μm pore size; ANPEL, Shanghai, China) before HPLC-MS analysis. The HPLC analytical conditions used were identical to that of cytokinin measurement, with a flow rate of 0.40 mL/min. Linear ion trap (LIT) and triple quadrupole (QQQ) scans were acquired on a triple quadrupole-linear ion trap mass spectrometer (QTRAP, Boston, USA), API 4500 Q TRAP LC/MS/MS system, equipped with an ESI Turbo Ion-Spray interface, based on the optimized declustering potential (DP) and collision energy (CE), and controlled by Analyst 1.6.3 software (AB Sciex, Singapore). The ESI source operation parameters were as follows: ion source, turbo spray; source temperature, 500°C; ion spray voltage (IS), 5,500 V; ion source gas I (GSI), 55.0 psi; gas II (GSII), 60.0 psi, curtain gas (CUR), 25.0 psi; and collision gas (CAD), high. Metabolite identification was carried out according to secondary spectral information, based on metabolite public databases, namely MassBank (http://www.massbank.jp/), KNAPSAcK (http://kanaya.naist.jp/KNApSAcK/), HMDB (http://www.hmdb.ca/), MoToDB (http://www.ab.wur.nl/moto/), and METLIN (http://metlin.scripps.edu/index.php/). Metabolite quantification was analyzed using the multiple reaction monitoring (MRM) mode of QQQ. Unsupervised principal component analysis (PCA) and supervised multiple regression orthogonal partial least-squares-discriminant analysis (OPLS-DA) were performed to visualize the metabolic alterations among experimental groups after mean centering and unit variance scaling. Metabolites with |log_2_ fold change| ≥1.0 and variable importance in projection (VIP) ≥1.0 were identified as the differential metabolites between *OsCKX2* OE rice and WT. Annotated metabolites were mapped to the Kyoto Encyclopedia of Genes and Genomes (KEGG) pathway database (http://www.kegg.jp/kegg/pathway.html/) to analyze pathway associations.

## Results

### Root-Specific Overexpression of *OsCKX2* Reduced Cytokinin Levels

**Figure 1:**
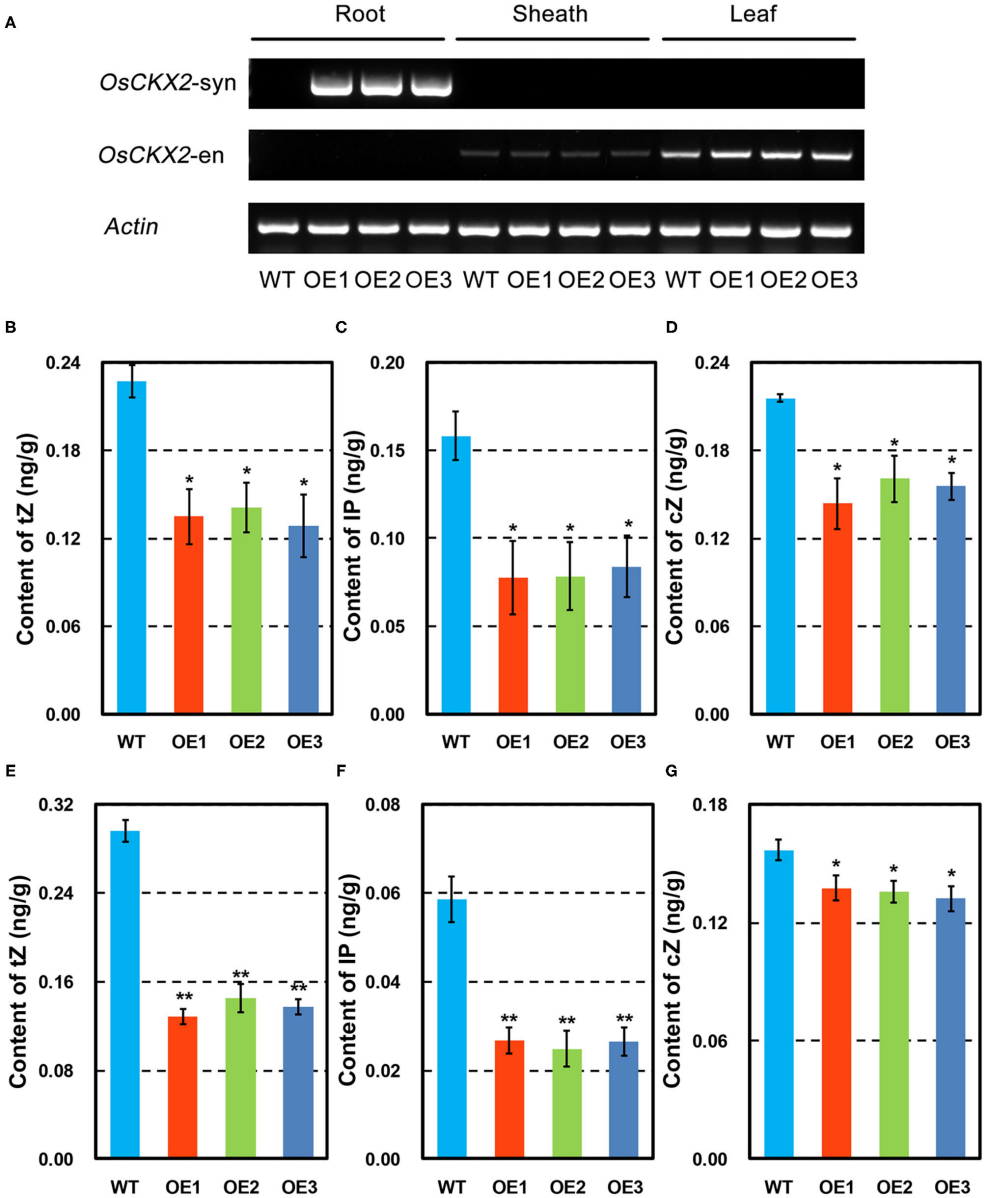
Characteristics of *OsCKX2* OE rice. Expression of synthetic and endogenous *OsCKX2* in root, sheath, and leaf by semi-qPCR **(A)**. Contents of cytokinins in different forms of tZ, IP, and cZ at seedling stage **(B–D)** and mature stage **(E–G)**. Data represents means ± SEM (*n* = 3). *P < 0.05; **P < 0.01.

The synthetic *OsCKX2* was driven by root-specific promoter *RCc3* to obtain *OsCKX2* OE rice (Supplementary Figure 1), and its expression was quite root-specific confirmed by semi-qPCR and qRT-PCR (Figure 1A and Supplementary Figure 2). Cytokinin measurement by UPLC-MS/MS showed significantly lower tZ, IP, and cZ levels in *OsCKX2* OE rice at seedling and mature stages (*P* < 0.05; *P* < 0.01) (Figures 1B–G). These results indicated that endogenous cytokinins were degraded resulted from root-specific overexpression of *OsCKX2*.

### Phenotypic Traits of *OsCKX2* OE Rice at Seedling Stage

**Figure 2:**
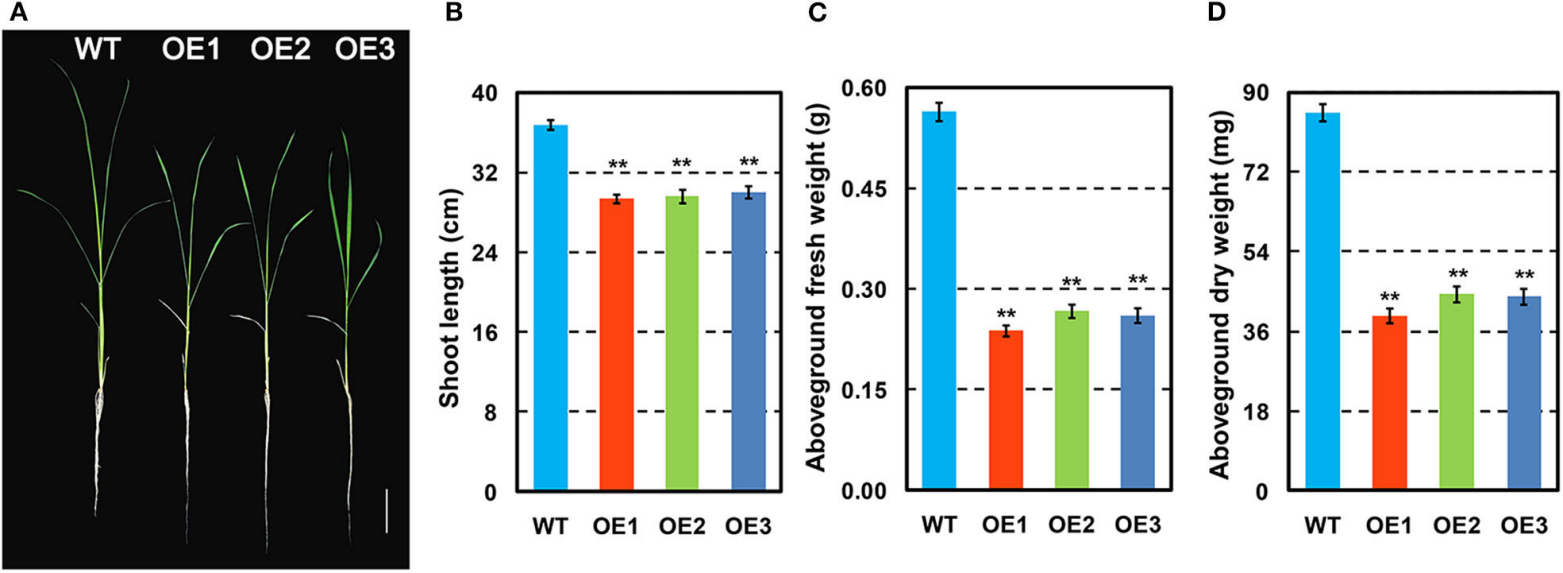
Phenotypes of *OsCKX2* OE rice and WT at seedling stage. Plant morphology at seedling stage **(A)**. Statistical analysis of shoot length **(B)**, aboveground fresh weight **(C)**, and aboveground dry weight **(D)**. Data represents means ± SEM (*n* = 15). **P < 0.01. Scale bars: 5 cm.

The phenotypes of *OsCKX2* OE rice and WT plants were evaluated at seedling stage. Compared to WT, *OsCKX2* OE rice had shorter shoots (Figures 2A,B) and reduced aboveground fresh and dry weight (Figures 2C,D). The traits shoot length, aboveground fresh weight, and aboveground dry weight of *OsCKX2* OE rice were 18.33–20.18%, 52.76–57.91%, and 48.12–53.80% lower than the WT plants, respectively. In the field, the growth of *OsCKX2* OE rice was retarded in the vegetative growth phase (Figure 5A). Overall, root-specific overexpression of *OsCKX2* significantly reduced shoot length and biomass at seedling stage.

### Phenotypic Traits of *OsCKX2* OE Rice at Mature Stage

**Figure 3:**
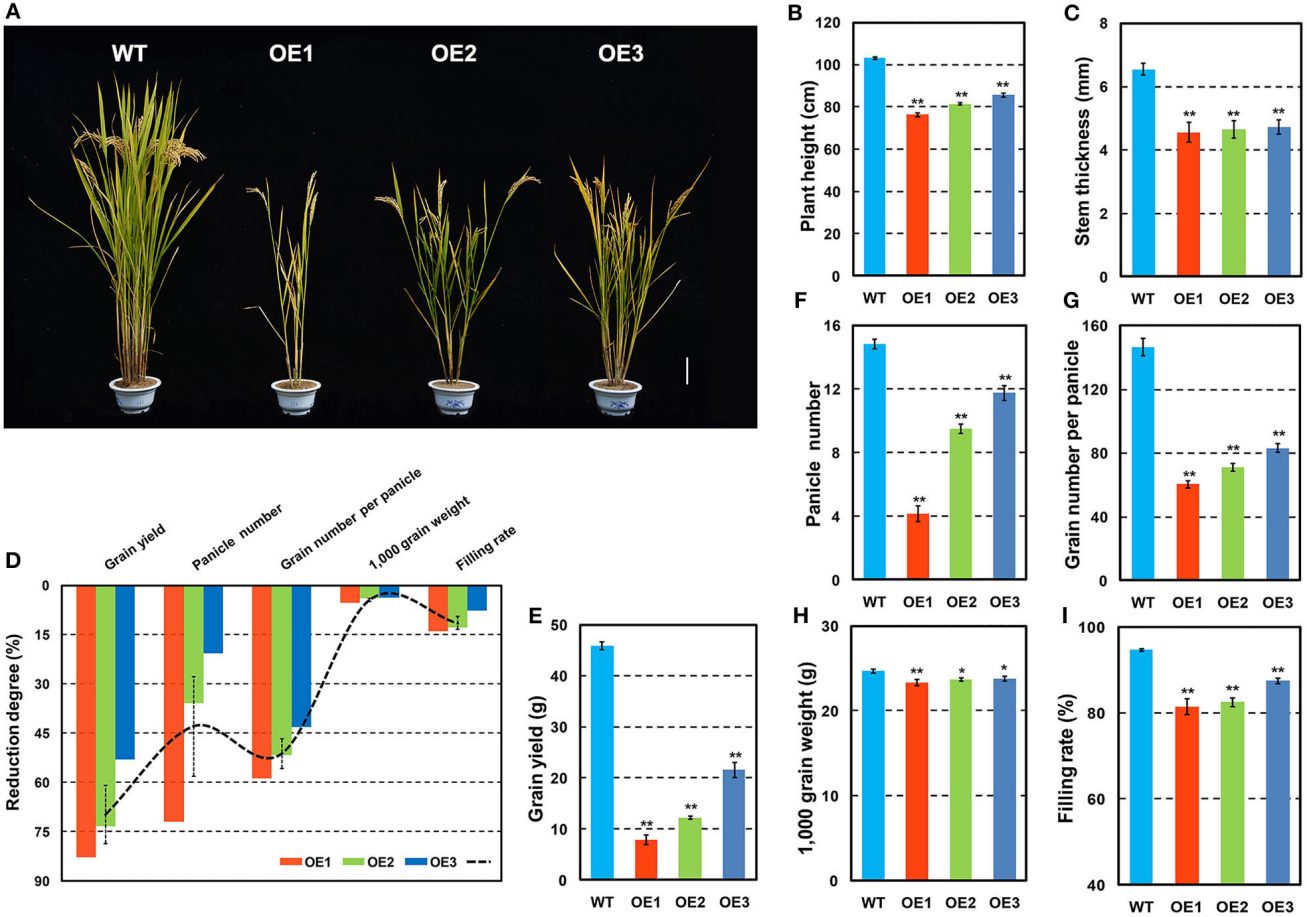
Phenotypes and yield related traits of *OsCKX2* OE rice and WT at mature stage. Plant morphology at mature stage **(A)**. Statistical analysis of plant height **(B)**, stem thickness **(C)**, reduction degree of yield related traits **(D)**, grain yield **(E)**, panicle number **(F)**, grain number per panicle **(G)**, 1,000 grain weight **(H)**, and filling rate **(I)**. Data represents means ± SEM (*n* = 15). **P < 0.01, *P < 0.05. Scale bars: 10 cm.

**Figure 4:**
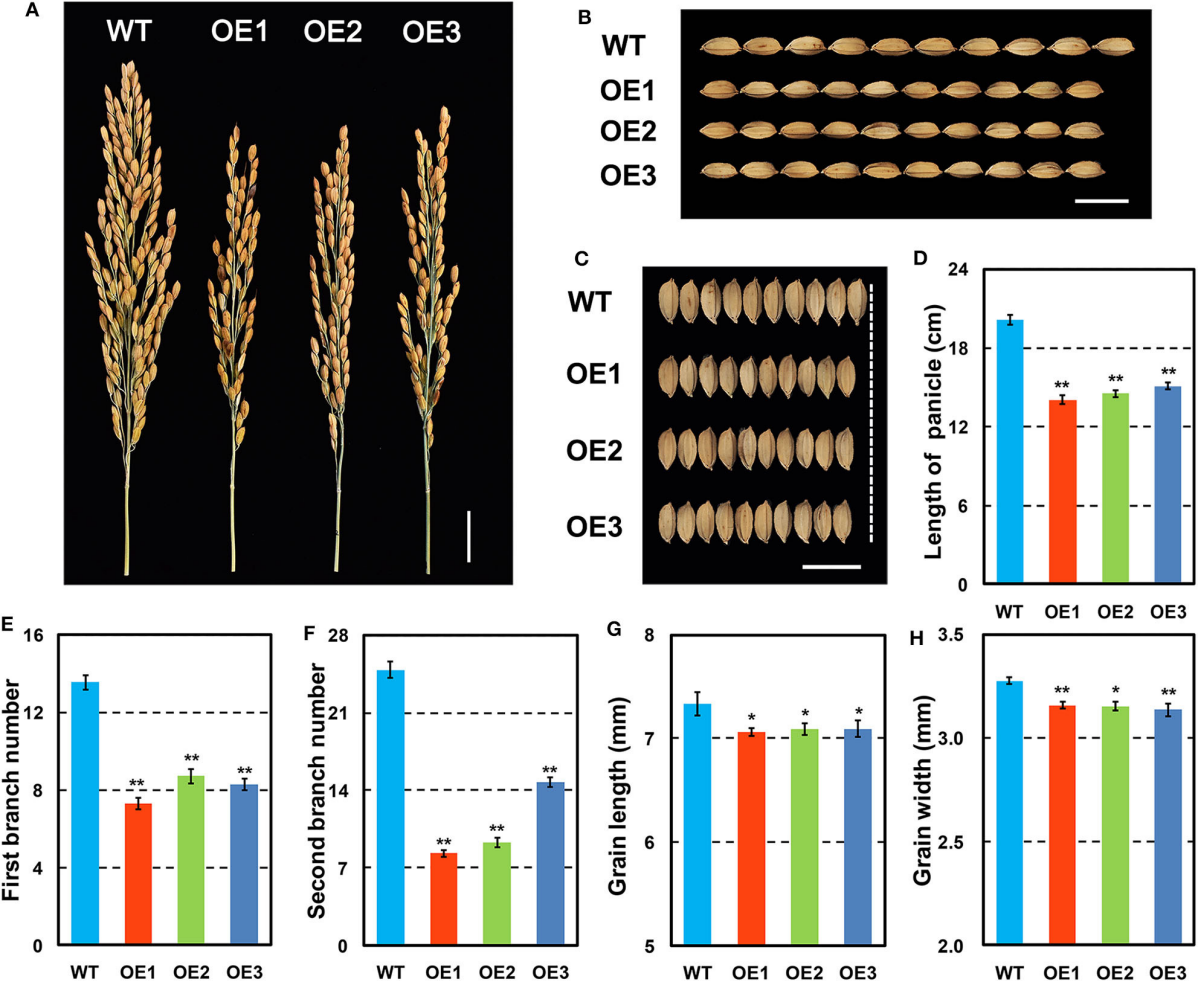
Detailed panicle and grain features of *OsCKX2* OE rice and WT. Phenotypes of panicles **(A)**, and grains **(B,C)**. Statistical analysis of length of panicle **(D)**, first branch number **(E)**, second branch number **(F)**, grain length **(G)**, and grain width **(H)**. Data represents means ± SEM (*n* = 15). **P < 0.01, *P < 0.05. Scale bars: 2 cm **(A)**, 1 cm **(B,C)**.

**Figure 5:**
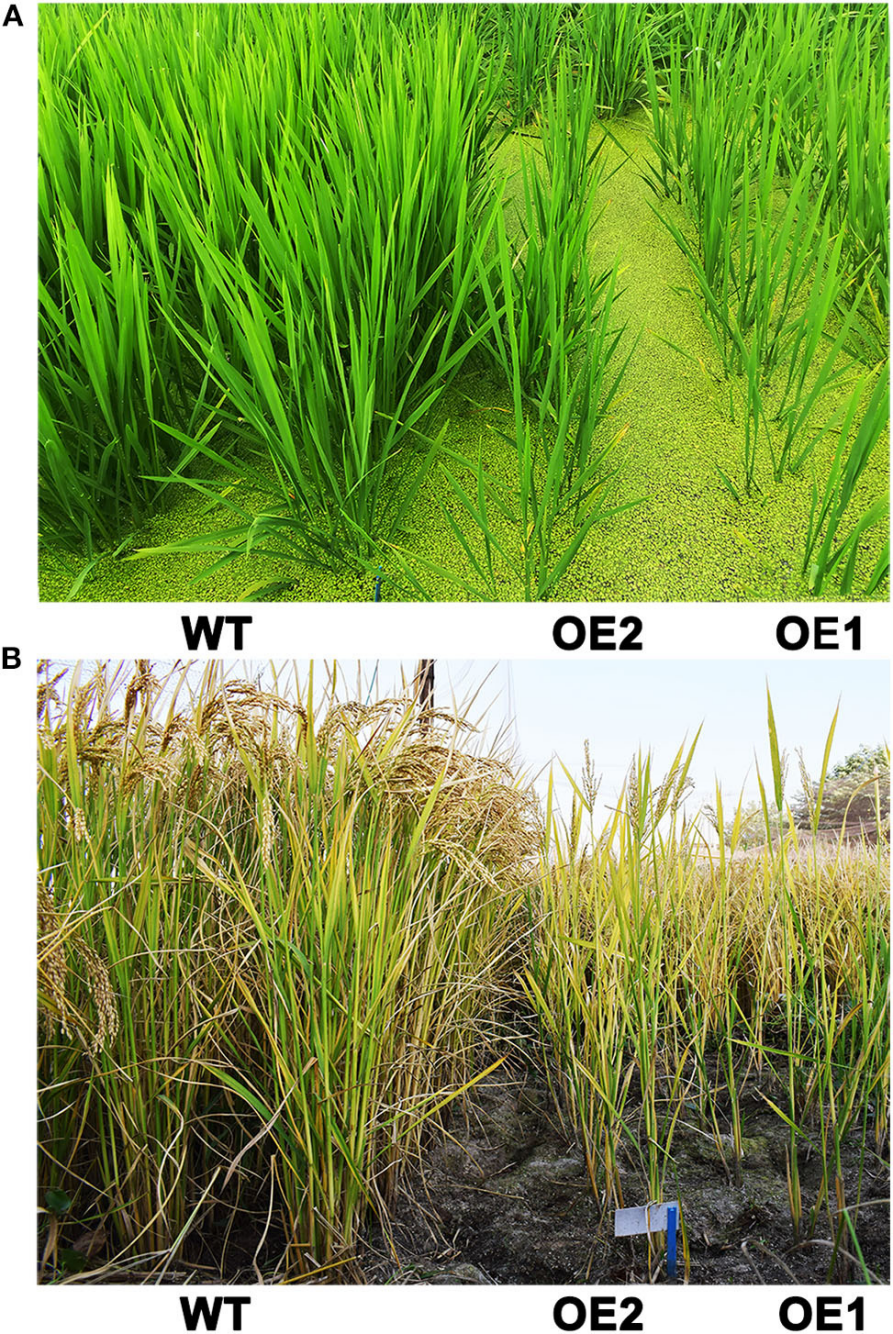
Phenotypes of *OsCKX2* OE rice and WT in vegetative growth phase **(A)** and at mature stage **(B)** under field condition.

*OsCKX2* OE rice exhibited reduced plant height and stem thickness at mature stage compared with WT (Figures 3A–C). Grain yield of *OsCKX2* OE rice was 53.04–82.94% lower than WT (Figure 3E). The traits panicle number, grain number per panicle, 1,000 grain weight, and filling rate of *OsCKX2* OE rice were 20.79–72.02%, 43.27–58.87%, 3.76–5.39%, and 7.62–14.00% lower, respectively (Figures 3F–I). Among these yield parameters, panicle number, and grain number per panicle mainly contributed to yield decline followed by filling rate (Figure 3D). *OsCKX2* OE rice produced smaller panicles and fewer first and second branches (Figures 4A,D–F). Besides, grains of *OsCKX2* OE rice were smaller than WT due to decreased grain length and grain width (Figures 4B,C,G,H). In the field, *OsCKX2* OE rice grew more weakly than WT (Figure 5B). In general, overexpression of *OsCKX2* reduced yield and multiple phenotypic traits at mature stage.

### Overview of RNA-seq Data Analysis

RNA-seq, using shoots of *OsCKX2* OE rice and WT at four to five leaf stage, resulted in approximately 52.35–71.46 million clean reads (Supplementary Table 3). The average rate of reads mapped to the rice reference genome was >95.00%, and the unique mapping rate ranged from 92.67 to 93.85% (Supplementary Table 3). Distribution statistics of reads on the gene showed that most of the reads (73.71–76.20%) were mapped to the coding sequence (CDS) (Supplementary Figure 3). In total, 1,743 DEGs were identified in the global transcriptional profiles. The volcano plot of DEGs showed that 58.86% was up-regulated, while 41.14% was down-regulated in *OsCKX2* OE rice (Supplementary Figure 4). The expression levels of several DEGs identified by RNA-seq were validated by qRT-PCR (Supplementary Figure 5).

### Down-Regulated DEGs Between *OsCKX2* OE Rice and WT

**Figure 6:**
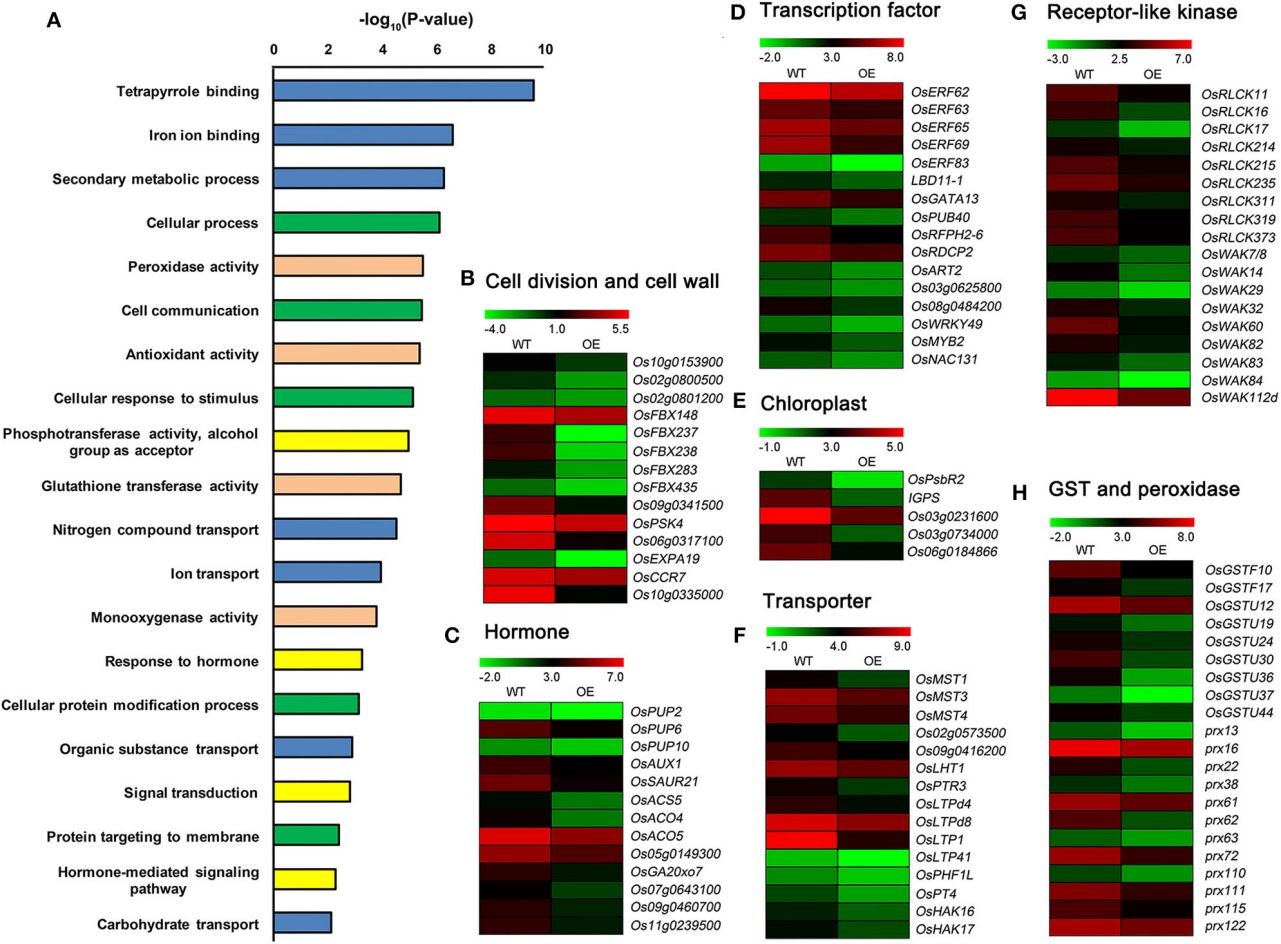
Detailed analysis of down-regulated DEGs between *OsCKX2* OE rice and WT. Terms clustering analysis of down-regulated DEGs **(A)**. Value in the X-coordinate is the corrected *P*-value by –log10. Expression levels of genes related to cell division and cell wall structure **(B)**, hormone **(C)**, transcription factor **(D)**, chloroplast formation **(E)**, transporter **(F)**, receptor-like kinase **(G)**, and GST and peroxidase **(H)** decreased in *OsCKX2* OE rice. The color legend indicates Log_2_ (FPKM) value.

GO enrichment analysis revealed that the down-regulated DEGs participated in cellular processes, hormone and signaling pathways, metabolic processes, substances transport, stress response processes, and regulation and protection function (Figure 6A and Supplementary Figure 6A). Many down-regulated DEGs, such as *Os10g0153900* encoding cyclin-dependent kinase, *Os02g0800500* and *Os02g0801200* encoding cyclin B, *OsFBX148, OsFBX237, OsFBX238, OsFBX283, OsFBX435*, and *Os09g0341500* encoding cyclin-like F-box domain containing proteins (FBXs), *OsPSK4* encoding phytosulfokines 4 precursor, *Os06g0317100* encoding glycine-rich cell wall structural protein, *OsCCR7* encoding cinnamoyl-CoA reductase to regulate lignin biosynthesis, *Os10g0335000* encoding dirigent protein in lignin biosynthesis, and *OsEXPA19* encoding expansin precursor (Figure 6B), were related to cell division or cell wall structure. A number of DEGs were related to transcription factors and hormone signaling, such as cytokinin, auxin, ethylene, and gibberellin (Figures 6C,D). Several other DEGs, such as *OsPsbR2, IGPS*, and *Os03g0231600* encoding chloroplast precursors, *Os03g0734000* and *Os06g0184866* encoding pentatricopeptide repeat domain containing proteins (PPRs), and genes encoding monosaccharide transporters (MSTs), peptide transporters, lipid transfer proteins (LTPs), and phosphate and potassium transporters, were involved in chloroplast development and nutrients transport (Figures 6E,F). Many down-regulated DEGs encoded receptor-like cytoplasmic kinases (RLCKs), wall-associated kinases (WAKs), glutathione-S-transferases (GSTs), and peroxidases (Figures 6G,H).

### Up-Regulated DEGs Between *OsCKX2* OE Rice and WT

**Figure 7:**
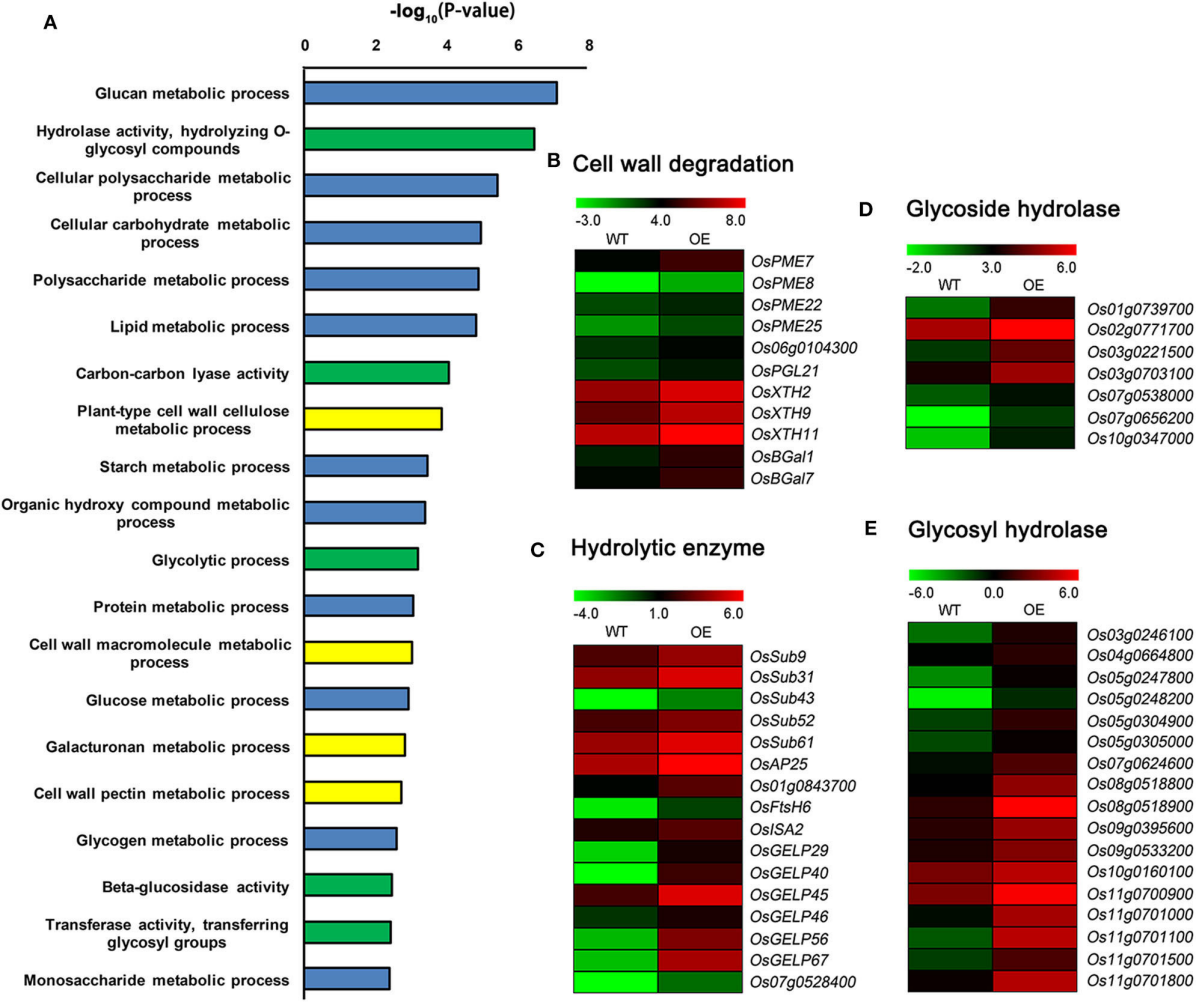
Detailed analysis of up-regulated DEGs between *OsCKX2* OE rice and WT. Terms clustering analysis of up-regulated DEGs **(A)**. Value in the X-coordinate is the corrected *P*-value by –log10. Expression levels of genes related to cell wall degradation **(B)**, hydrolytic enzyme **(C)**, glycoside hydrolase **(D)**, and glycosyl hydrolase **(E)** increased in *OsCKX2* OE rice. The color legend indicates the Log_2_ (FPKM) value.

GO enrichment analysis revealed that the up-regulated DEGs were clustered in metabolic processes, response processes, cellular processes, molecular regulation, and hormone and signaling pathways. The GO term metabolic processes were highly enriched (Supplementary Figure 6B). The prominent categories of metabolic processes included cell wall components metabolic process, nutrient substances metabolic process and hydrolase activity (Figure 7A). Many up-regulated DEGs, such as genes encoding pectinesterase (PME), polygalacturonase (OsPGL21), xyloglucan endotransglycosylase/hydrolase (XTH), and β-galactosidase (BGal), were related to cell wall degradation (Figure 7B). Several other up-regulated genes, such as genes encoding subtilisin-like protease, aspartic protease (OsAP25), FtsH protease (OsFtsH6), β-amylase (OsISA2), and GDSL esterase/lipase (GELP), were involved in nutrient substances catabolic processes (Figure 7C). Moreover, numerous glycoside and glycosyl hydrolase genes were up-regulated in *OsCKX2* OE rice (Figures 7D,E).

### Metabolite Variation Between *OsCKX2* OE Rice and WT

**Figure 8:**
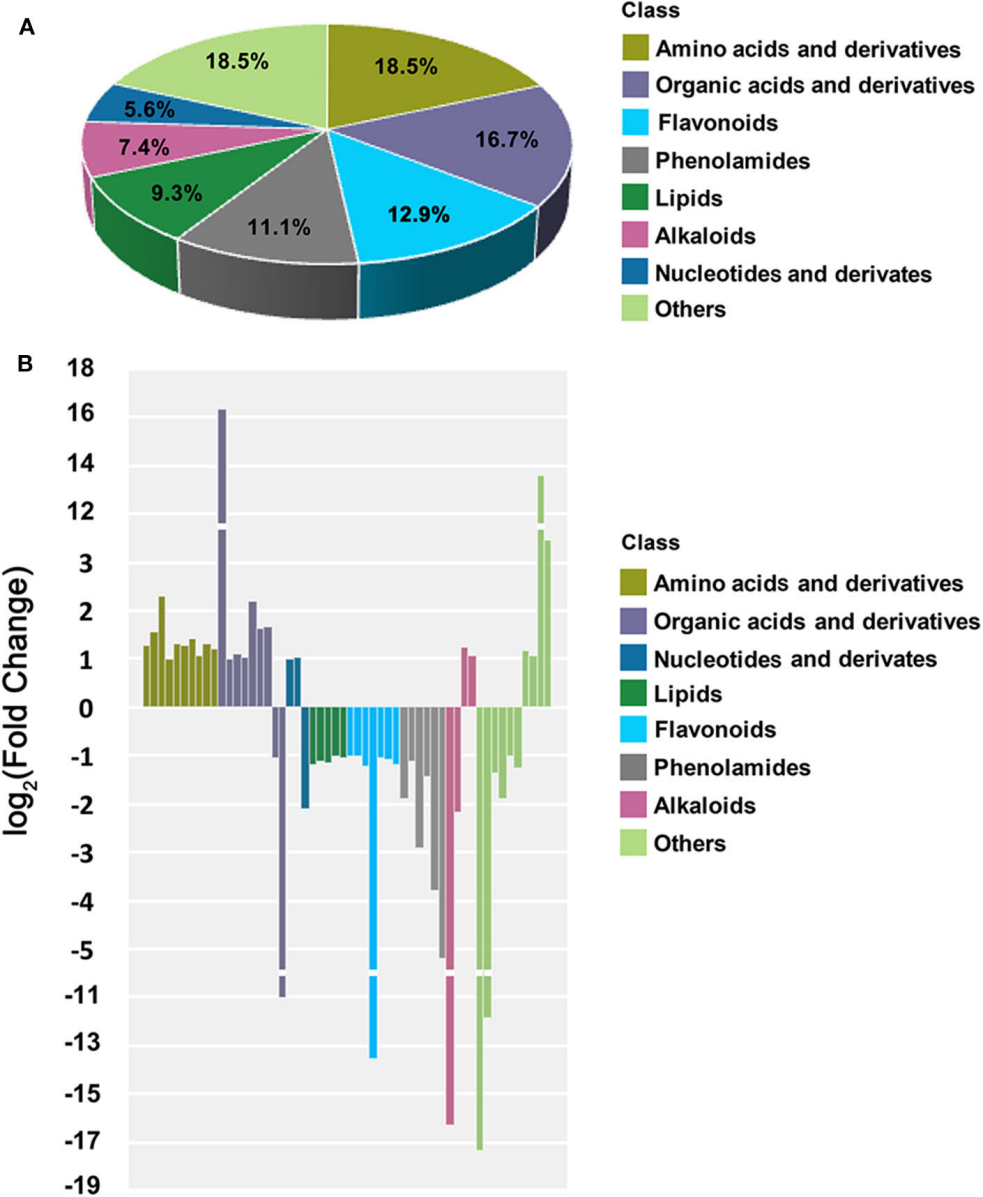
Overview of differential metabolites in *OsCKX2* OE rice compared to WT. Categorization of differential metabolites **(A)**. Histogram of metabolites with significantly altered concentration **(B)**. X-coordinate indicates the classification of differential metabolites, and Y-coordinate represents the magnitude of concentration difference.

A total of 778 metabolites were detected by UPLC-MS/MS in the shoots of *OsCKX2* OE rice and WT. A total of 54 metabolites showed differential accumulation in *OsCKX2* OE rice compared with WT. Amino acids and derivatives, organic acids and derivatives, flavonoids, phenolamides, lipids, alkaloids, nucleotides and derivatives, and other metabolites accounted for 18.5, 16.7, 12.9, 11.1, 9.3, 7.4, 5.6, and 18.5% of the differential metabolities, respectively (Figure 8A). Many differential primary metabolites, including all amino acids and derivatives, 78% of organic acids and derivatives, and 67% of nucleotides and derivatives, were present at a greater concentration in *OsCKX2* OE rice than WT. On the contrary, few differential primary metabolites, including all lipids, 22% of organic acids and derivatives, and 33% of nucleotides and derivatives, were present at a lower concentration in *OsCKX2* OE rice (Figure 8B).

### Integrated Transcriptomic and Metabolic Data

**Figure 9:**
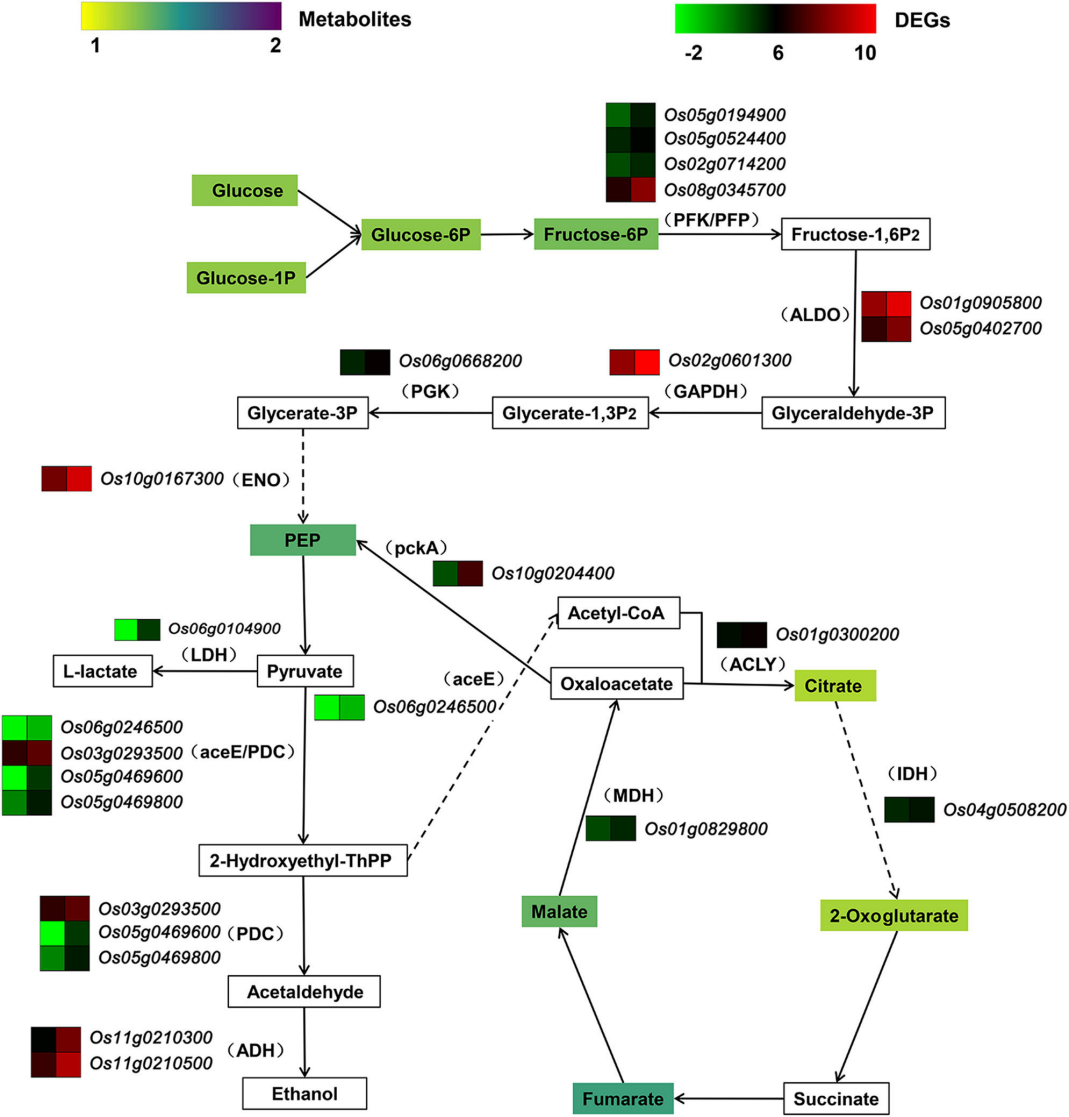
Pathway of carbohydrate catabolism combined transcriptome and metabolome. Rectangle in the pathway indicates metabolite. Legend of metabolite shows fold change in *OsCKX2* OE rice compared to WT. Bracket indicates enzyme corresponding to DEG. Gene heatmap shows the value of Log_2_ (FPKM) in WT (left panel) and *OsCKX2* OE rice (right panel). Solid arrow represents direct metabolism step. Broken arrow indicates indirect enzymatic reaction.

An integrated analysis of transcriptomic and metabolomic data revealed that the up-regulated DEGs and the elevated metabolites were related to glycolysis and tricarboxylic acid cycle (TCA), mainly responsible for carbohydrate catabolism (Figure 9). Glucose, the initial reactant of glycolysis, was present at a greater concentration in *OsCKX2* OE rice. Accompanied by the up-regulation of several DEGs, such as genes encoding 6-phosphofructokinase (PFK), diphosphate-dependent phosphofructokinase (PFP), fructose-bisphosphate aldolase (ALDO) and glyceraldehyde 3-phosphate dehydrogenase (GAPDH), phosphoenolpyruvic acid (PEP) content was increased in *OsCKX2* OE rice. Some genes related to anaerobic glycolysis, which produced lactate and ethanol, were up-regulated. The expression of the gene encoding pyruvate dehydrogenase E1 component (aceE), which promoted the synthesis of acetyl-CoA, was up-regulated. In *OsCKX2* OE rice, genes encoding ATP citrate (pro-S)-lyase (ACLY), isocitrate dehydrogenase (IDH), and malate dehydrogenase (MDH) were up-regulated, and citrate, 2-oxoglutarate, fumarate and malate contents (of TCA pathway) were increased to varying degrees.

## Discussion

Regulation of endogenous cytokinin levels in addition to exogenous application of cytokinins to regulate and improve plant growth has become a topic for plant developmental research. Researches on *CKX* gene family, which encodes cytokinin-degrading enzymes to regulate cytokinin levels, help to understand cytokinin regulatory roles and signaling pathway. Increase in cytokinin contents by reducing *OsCKX2* transcription levels enhances meristem activity, elevates grain and tiller number, and ultimately increases rice grain production (Ashikari et al., 2005; Yeh et al., 2015), with no effect on plant height (Joshi et al., 2018). Therefore, we aim at decrease in cytokinin levels via increasing the transcription levels of *OsCKX2* specifically in roots to study the influence on rice growth. Overexpression of *OsCKX2* driven by the root-specific promoter *RCc3* reduced endogenous cytokinin contents, including tZ, IP, and cZ, resulted in reduced plant height with weaker roots (Supplementary Figure 7), thinner stems, fewer tillers, smaller panicles, lower filling rate, and smaller grains. Among the different type ctokinins, tZ-type are predominantly synthesized in roots and are necessary for normal shoot development (Matsumoto-Kitano et al., 2008; Kudo et al., 2010; Ko et al., 2014; Zhang et al., 2014). Overexpression of *OsCKX2* in roots reduced cytokinin levels, especially tZ, led to retarded growth at seedling and mature stages. These results implied that overexpression of *OsCKX2* in roots have disrupted the dynamic equilibrium of cytokinins in the whole plants.

Cytokinins are essential signaling molecules that regulate cell division and proliferation. RNA-seq data analysis revealed that cell cycle-related genes, such as cyclin-dependent kinase gene, cyclin B genes, several *OsFBX* genes and *OsPSK4*, were down-regulated in *OsCKX2* OE rice. Studies have shown that cytokinins regulate the expression of cyclin-dependent kinases and cyclins, which play crucial roles in plant cell cycle progression (Guo et al., 2007; Schaller et al., 2014). The F-box genes (*FBXs*) can affect plant cell cycle and participate in the control of cell proliferation (Boycheva et al., 2015). Phytosulfokine-α, a peptide plant growth factor, encoded by *PSK* gene, is essential for cell proliferation (Yang et al., 2000). The cellular processes regulate plant cell wall integrity, and the coordination between cell cycle and plant cell wall integrity is necessary for normal plant development (Gigli-Bisceglia and Hamann, 2018). In this study, overexpression of *OsCKX2* decreased the expression of cell wall-related genes (*Os06g0317100, OsEXPA19, OsCCR7*, and *Os10g0335000*), while it increased the expression of genes encoding plant cell wall-degrading enzymes, such as *PME, OsPGL21, XTH*, and *BGal*. Cell wall damage induces high expression of *CKX* genes and accelerates the degradation rate of cytokinins that in turn reduces the expression of cell cycle genes (Gigli-Bisceglia et al., 2018). Down-regulated genes related to cell wall structure and up-regulated genes related to cell wall degradation may disrupt cell wall stability in *OsCKX2* OE rice. Taken together, the overexpression of *OsCKX2* reduced cell cycle activity and cell wall stability in rice that subsequently led to retarded morphogenesis.

Crosstalk between cytokinins and other phytohormones is crucial for plant growth and development. Cytokinins are known to control the activity and function of shoot and panicle meristems interacting with auxin and gibberellin signaling (Durbak et al., 2012; Wu et al., 2016). Cytokinin/ethylene crosstalk plays a key role in seed germination and early seedling development (Zdarska et al., 2015). In *OsCKX2* OE rice, many genes related to hormone biosynthesis, transport, and signal transduction, including cytokinin and auxin transporters, auxin-responsive genes, gibberellin 20-oxidase genes, putative gibberellin receptor and ethylene biosynthesis genes, were down-regulated. Additionally, transcription factors participate in phytohormone signal pathway. The expression of transcription factors, such as *OsERF* and *OsGATA13*, was down-regulated In *OsCKX2* OE rice. AP2/ERF transcription factor controls cytokinin-triggered shoot regeneration origination (Banno et al., 2001). Cytokinin GATA transcription factor has been reported to control chloroplast development and plant architecture in rice (Hudson et al., 2013). Taken together, the abnormal expression of phytohormone-related genes and transcription factors may have led to poor growth of *OsCKX2* OE rice.

Cytokinins have been proven to be involved in chloroplast development (Cortleven and Schmülling, 2015). RNA-seq data revealed that several nuclear genes encoding PsbR, chloroplast precursors, and PPR proteins were down-regulated in *OsCKX2* OE rice. PsbR regulates the formation of oxygen-evolving complex of photosystem II (Allahverdiyeva et al., 2013), and PPR proteins participate in plastid RNA processing in chloroplasts (Legen et al., 2018; Zhang et al., 2019). Cytokinins also influence nutrient uptake and translocation in plants. In this study, the transcriptional levels of transporters, such as monosaccharide transporters, peptide transporters and phosphate and potassium transporters, were lower in *OsCKX2* OE rice. Cytokinins enhanced mRNA accumulation of hexose transporter genes to supply carbohydrates to sink tissues (Roitsch and Ehneß, 2000). Studies have reported that cytokinins involve in amino acid transport and phosphate and potassium homeostasis by controlling the expression of related transporters or signaling genes (Wang et al., 2006; Nam et al., 2012; Ninan et al., 2019). Altogether, these findings indicate that chloroplast genesis and nutrient transport in *OsCKX2* OE rice may be defective, which were detrimental to the assimilation, translocation, and distribution of nutrients.

Carbohydrates provide energy for life activities via catabolism and also get stored in sink organs to build up plant biomass, which maintains a precise equilibrium partitioning of carbohydrates during plant life cycle (Zakhartsev et al., 2016; Julius et al., 2018). The disability to convert photosynthates into starch resulted in higher soluble carbohydrate levels, higher respiration rate and retarded growth in *Arabidopsis thaliana* (Caspar et al., 1985). The integrated transcriptome and metabolome data identified that many genes related to glycolysis and TCA cycle were significantly up-regulated and some intermediate metabolites associated with carbohydrate catabolism were accumulated in *OsCKX2* OE rice. In addition, numerous DEGs encoding hydrolytic enzymes, including subtilisin-like proteases, GDSL esterase/lipases, and glycoside and glycosyl hydrolases, showed elevated expression in *OsCKX2* OE rice, which may have accelerated the degradation of biomass components. The abundances of amino acids, organic acids, and their derivatives also increased in *OsCKX2* OE rice. In general, the excessive carbohydrate catabolic activity and hydrolytic activity weakened the normal biomass accumulation, which ultimately inhibited the growth of *OsCKX2* OE rice.

## Conclusion

The present study indicated that root-source cytokinins regulated the growth and development of rice. Reduced cytokinin levels by overexpressing *OsCKX2* specifically in roots resulted in dwarfing, lower biomass, fewer tillers, smaller panicles and grains, lower filling rate, and reduced yield in rice. Transcriptome and metabolome analysis revealed that *OsCKX2* overexpression affected cell cycle activity, cell wall structure, phytohormone and transcription factor signaling, chloroplast development and nutrient translocation, and nutrient degradation, which led to poor growth and development during the entire life cycle. This study broadens the understanding on the function of root-source cytokinins, and provides a basis for changing endogenous cytokinins by overexpression of *OsCKX2* specifically in root to regulate the biomass and yield in rice.

## Data Availability Statement

RNA-seq data has been uploaded to the National Center for Biotechnology Information (NCBI) Sequence Read Archive (SRA) database, under accession BioProject ID: PRJNA646215. The project information can be accessible with the following link (http://www.ncbi.nlm.nih.gov/bioproject/646215).

## Author Contributions

JL and QZ designed the project. HY performed experiment. HS, XJ, and CL investigated agronomic traits and contributed to analyze the data. HY and JL wrote the paper. All authors contributed to the article and approved the submitted version.

## Conflict of Interest

The authors declare that the research was conducted in the absence of any commercial or financial relationships that could be construed as a potential conflict of interest.

## Supplementary Material

Supplementary Figure 1 Vector diagram of *OsCKX2* OE rice. *OsCKX2* was driven by root-specific promoter *RCc3*.

Supplementary Figure 2 Relative expression levels of the synthetic *OsCKX2* gene in *OsCKX2* OE rice under KT (0.1 mM) treatment.

Supplementary Figure 3 Statistical distribution of reads on gene structure in *OsCKX2* OE rice and WT. **(A–F)** CDS, coding sequence; UTR, untranslated region; TSS, transcription start site; TES, transcription end Site.

Supplementary Figure 4 Volcano Plot of DEGs between *OsCKX2* OE rice and WT. Red dot represents up-regulated DEG. Green dot represents down-regulated DEG.

Supplementary Figure 5 Verification of RNA-seq data by qRT-PCR. Down-regulated DEGs **(A)** and up-regulated DEGs **(B)** in *OsCKX2* OE rice compared to WT. Data represents means ± SEM. ^**^*P* < 0.01, ^*^*P* < 0.05.

Supplementary Figure 6 GO enrichment of down-regulated DEGs **(A)** and up-regulated DEGs **(B)**. The dot represents GO term. The size of dot indicates gene number clustered in the GO term.

Supplementary Figure 7 Root morphology of *OsCKX2* OE rice. Statistical analysis of root number **(A)**, root thickness **(B)**, root length **(C)**, underground fresh weight **(D)**, and underground dry weight **(E)**. Data represents means ± SEM (n = 15). ^**^*P* < 0.01.

Supplementary Table 1 The endogenous gene, synthetic gene, and protein sequences of *OsCKX2*.

Supplementary Table 2 List of primers used in this study.

Supplementary Table 3 Summary of RNA sequencing and mapping using the rice genome (*Oryza sativa*) as reference.
