# Corrigendum: Phenotypic, Transcriptomic, and Metabolomic Signatures of Root-Specifically Overexpressed *OsCKX2* in Rice

**DOI:** 10.3389/fpls.2020.641990

**Published:** 2021-01-19

**Authors:** Huimin Yan, Hongzheng Sun, Xueying Jia, Chuanwei Lv, Junzhou Li, Quanzhi Zhao

**Affiliations:** Henan Key Laboratory of Rice Biology, Collaborative Innovation Center of Henan Grain Crops, Henan Agricultural University, Zhengzhou, China

**Keywords:** *OsCKX2*, cytokinin, transcriptome, metabolome, root-specific expression

In the original article, there was a mistake in Figure 1 as published. The corrected Figure 1 appears below.

**Figure 1:**
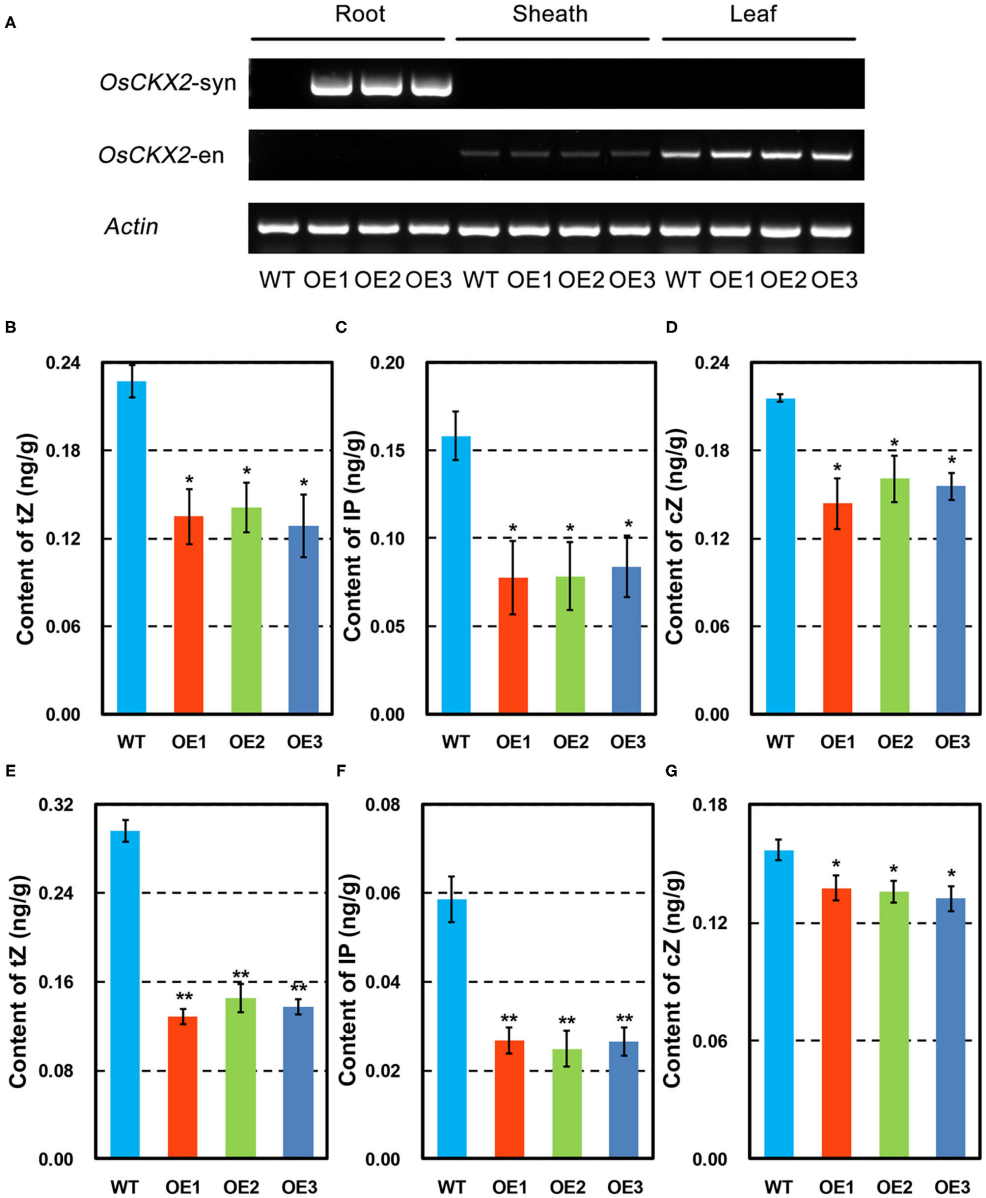
Characteristics of *OsCKX2* OE rice. Expression of synthetic and endogenous *OsCKX2* in root, sheath, and leaf by semi-qPCR **(A)**. Contents of cytokinins in different forms of tZ, IP, and cZ at seedling stage **(B–D)** and mature stage **(E–G)**. Data represents means ± SEM (*n* = 3). ^*^P < 0.05, ^**^P < 0.01.

In the original article, there was a mistake in Supplementary Table 2 as published. The corrected Supplementary Table 2 appears below.

**Supplementary Table 2:** List of primers used in this study.

**Name**	**Forward primer (5^**′**^-3^**′**^)**	**Reverse primer (5^**′**^-3^**′**^)**
*OsCKX2-syn*	GAGCAGTGCGTCGTTCCT	GCTTGCCCTCAAAAGTGC
*OsCKX2-en*	TTCGTCCGCCTCCTTCCT	CGACGCGCGCAGCAGCGC
*Actin*	GGAAGTACAGTGTCTGGATTGGAG	TCTTGGCTTAGCATTCTTGGGT
*OsPSK4*	GCCGTGCTGCTGATTTTC	GTGATGCTGCTGGGTGTAGA
*OsPUP6*	TCCCTGATGCAGCTCACGTT	TCGCCCTTCTTGTACCCGTC
*LBD11-1*	CCAGAACCAAGTCTCCCAGC	TCCACATGGACTCTTTCTTGAGG
*OsPsbR2*	GACCGAACCTGAAAGACGGT	CCAGCCAGCAGAATTCCAGA
*OsMST1*	TGACGTTCTCGGTGGTCATC	TAGACGCAGTACTCGTTCCC
*OsPME22*	ACTCGCTTCGCCAGTTCTAC	CTGGGGTGTATCAGGCTGTC
*OsPGL21*	AACTTGGCAGGGAGGTTCAG	CATGCAAAATGGGCAGGCTT
*OsAP25*	CCGAACTACACGTTCGGGTG	AGCGAGCCGGAGAAGTAGTA
*OsSub31*	GCTTACAGCGCCATGGAAAG	CCGGAGTAGTTCTTGCCGTT
*OsBGal1*	AGGAACCATCCGTCAACGAC	AGTCACAGTTGGATCGGCAG

The authors apologize for this error and state that this does not change the scientific conclusions of the article in any way. The original article has been updated.

