# Supplementary figures and images for: Phenotypic, Transcriptomic, and Metabolomic Signatures of Root-Specifically Overexpressed OsCKX2 in Rice
Source: Front Plant Sci. 2021 Jan 20;11:575304. doi: 10.3389/fpls.2020.575304 (PMC7719687; doi:10.3389/fpls.2020.575304)

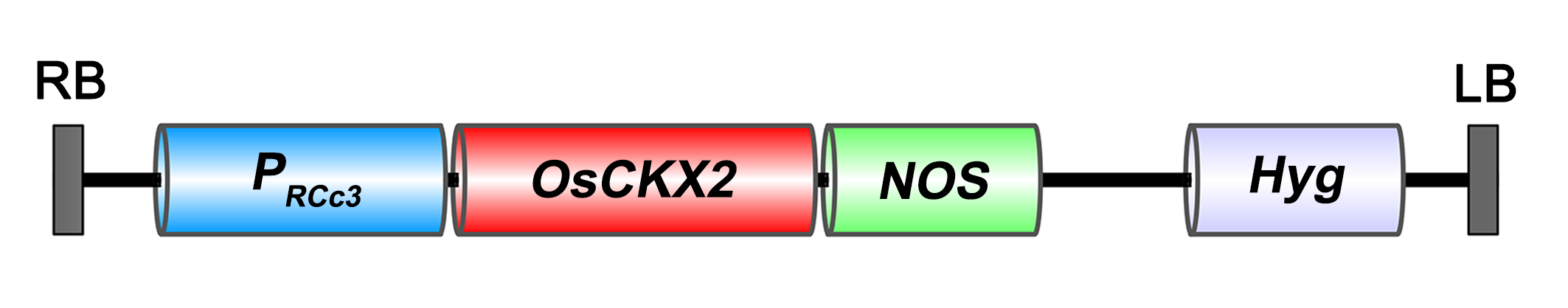

Supplement: Supplementary Figure 1 — Vector diagram of OsCKX2 OE rice. OsCKX2 was driven by root-specific promoter RCc3. [file Image_1.TIF]

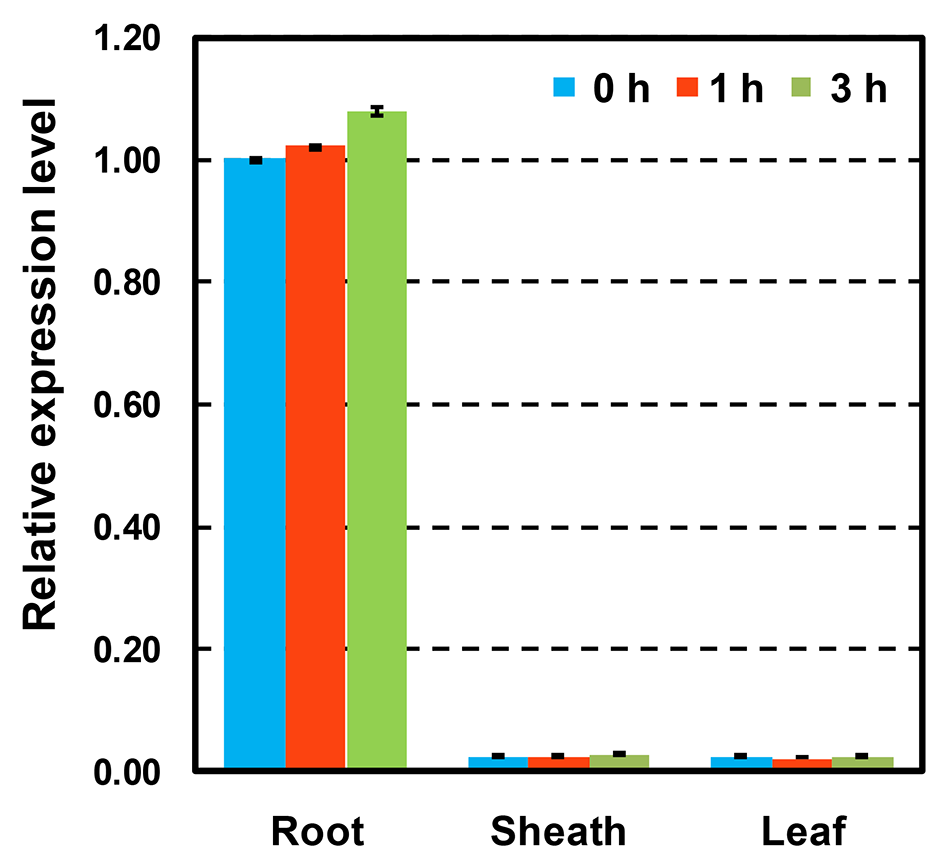

Supplement: Supplementary Figure 2 — Relative expression levels of the synthetic OsCKX2 gene in OsCKX2 OE rice under KT (0.1 mM) treatment. [file Image_2.TIF]

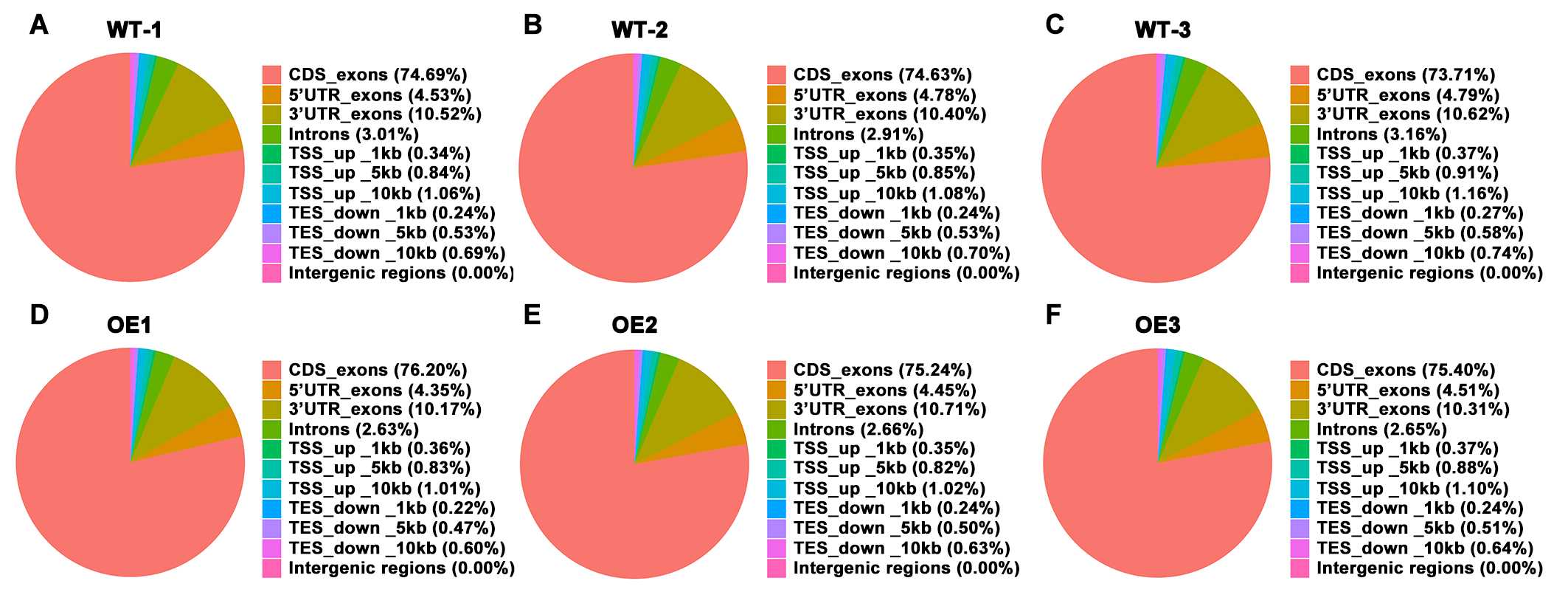

Supplement: Supplementary Figure 3 — Statistical distribution of reads on gene structure in OsCKX2 OE rice and WT. (A–F) CDS, coding sequence; UTR, untranslated region; TSS, transcription start site; TES, transcription end Site. [file Image_3.TIF]

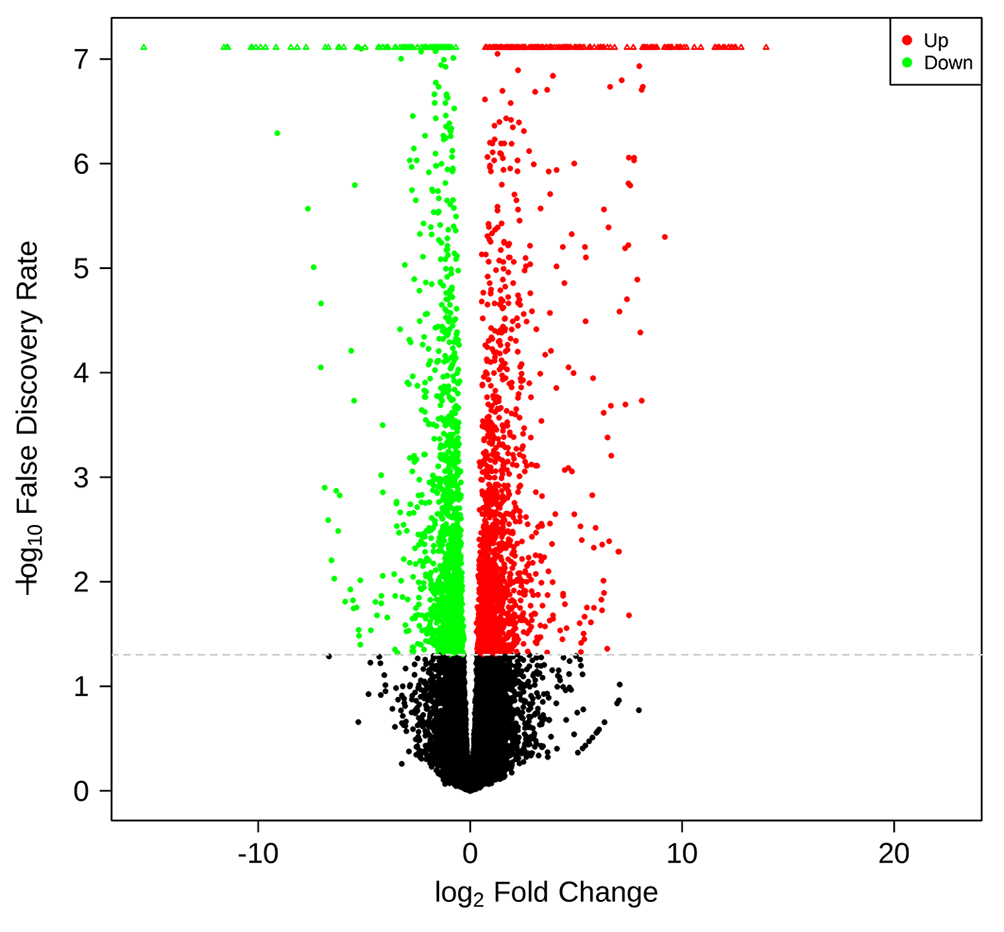

Supplement: Supplementary Figure 4 — Volcano Plot of DEGs between OsCKX2 OE rice and WT. Red dot represents up-regulated DEG. Green dot represents down-regulated DEG. [file Image_4.TIF]

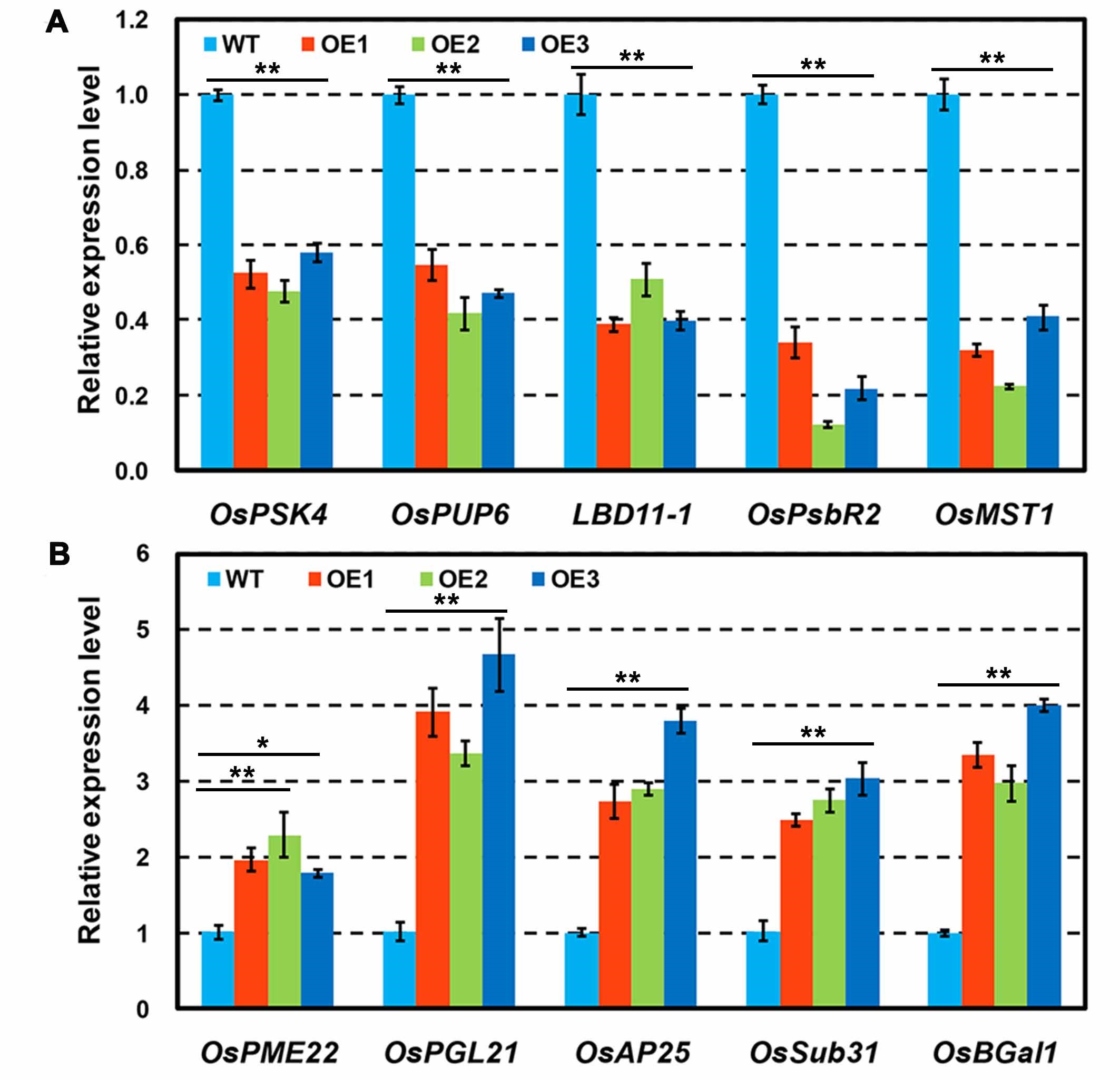

Supplement: Supplementary Figure 5 — Verification of RNA-seq data by qRT-PCR. Down-regulated DEGs (A) and up-regulated DEGs (B) in OsCKX2 OE rice compared to WT. Data represents means ± SEM. **P < 0.01, *P < 0.05. [file Image_5.jpg]

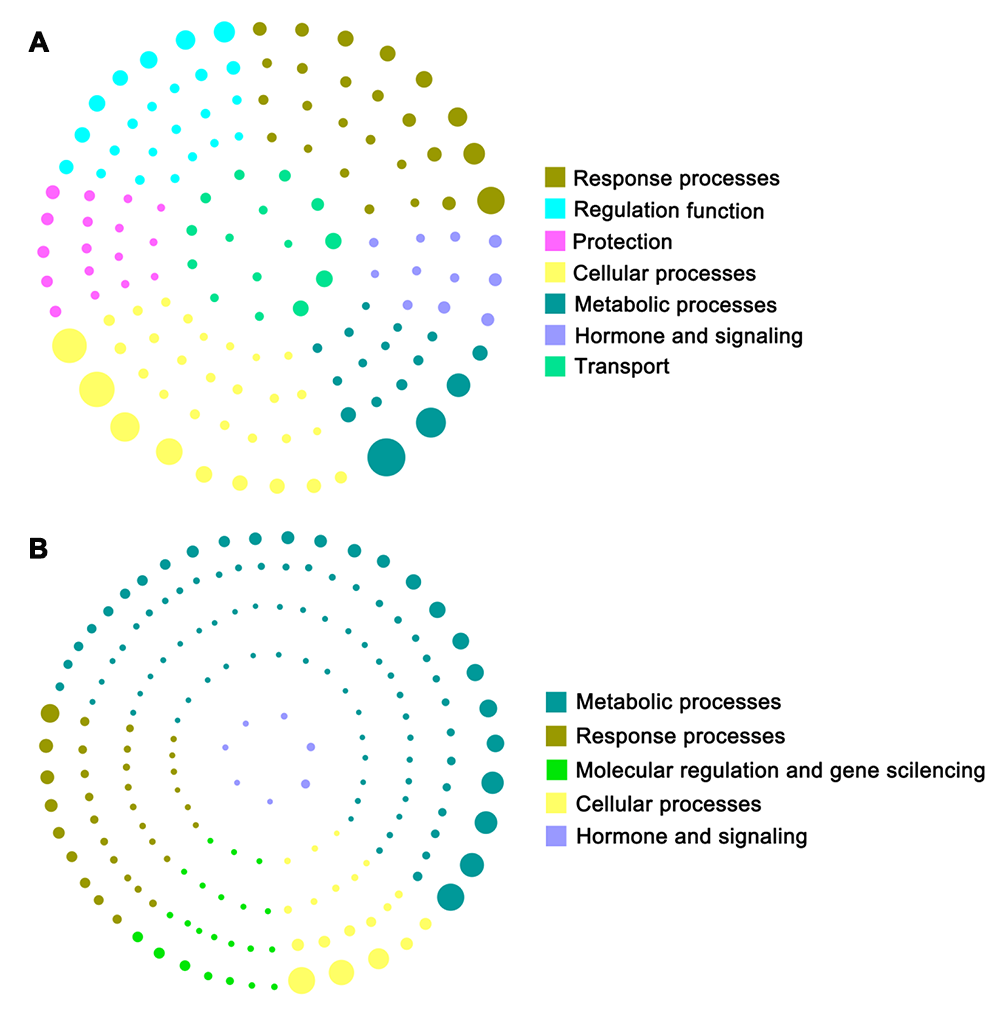

Supplement: Supplementary Figure 6 — GO enrichment of down-regulated DEGs (A) and up-regulated DEGs (B). The dot represents GO term. The size of dot indicates gene number clustered in the GO term. [file Image_6.TIF]

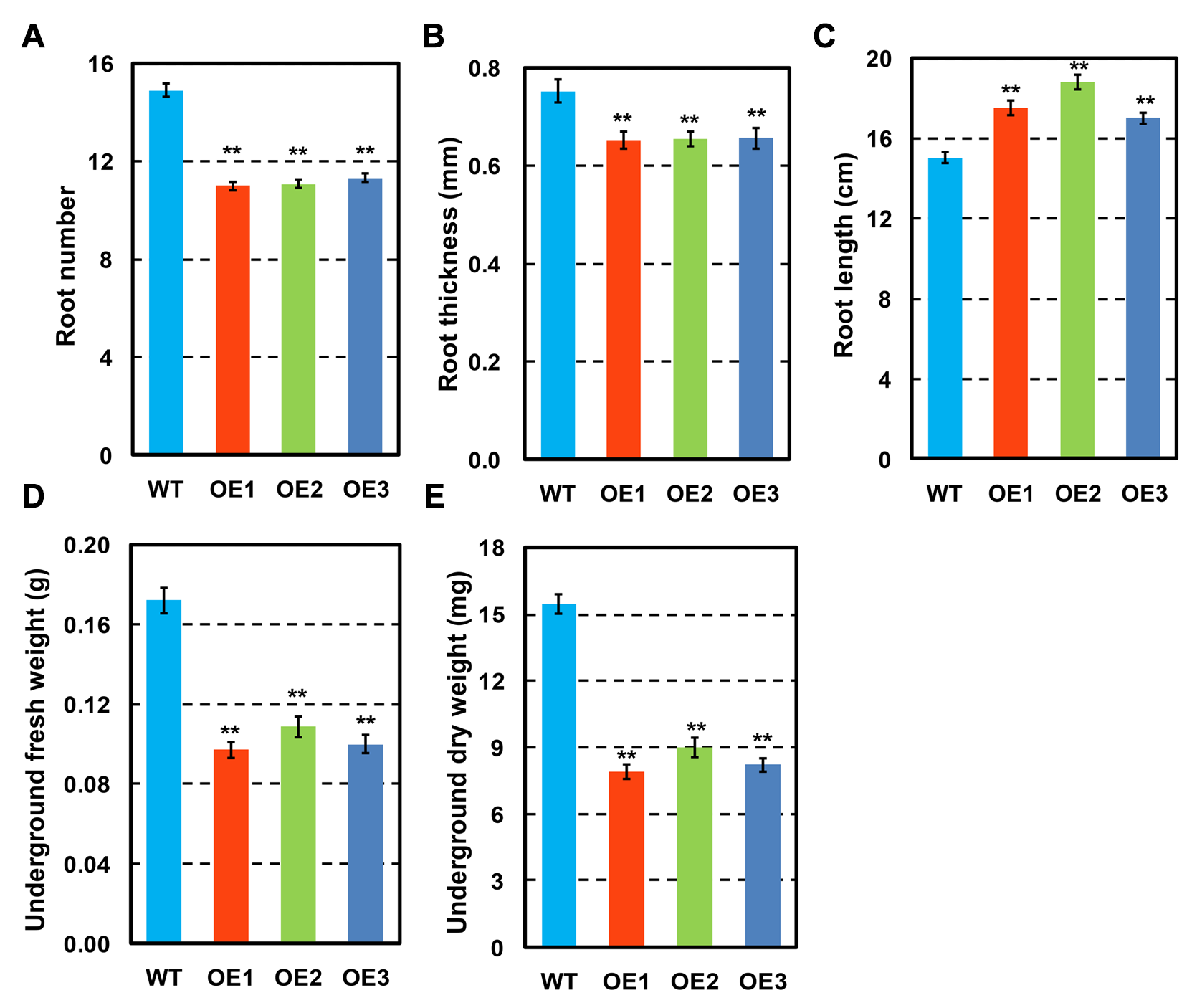

Supplement: Supplementary Figure 7 — Root morphology of OsCKX2 OE rice. Statistical analysis of root number (A), root thickness (B), root length (C), underground fresh weight (D), and underground dry weight (E). Data represents means ± SEM (n = 15). **P < 0.01. [file Image_7.TIF]
