# Supplementary material for: Phenotypic, Transcriptomic, and Metabolomic Signatures of Root-Specifically Overexpressed OsCKX2 in Rice
Source: Front Plant Sci. 2021 Jan 20;11:575304. doi: 10.3389/fpls.2020.575304 (PMC7719687; doi:10.3389/fpls.2020.575304)
Supplement: Supplementary Table 1 — The endogenous gene, synthetic gene, and protein sequences of OsCKX2. [file Table_1.DOC]

**Table S1. The endogenous gene, synthetic gene, and protein sequences of *OsCKX2*.**

| **Sequences** |
| --- |
| **Endogenous gene sequence**  ATGAAGCAAGAGCAGGTCAGGATGGCAGTGCTCCTCATGCTCAACTGCTTCGTCAAGGCCACGGCGCCGCCGCCATGGCCGCCGTCGGCTTCGTCCGCCTCCTTCCTCGACGACCTCGGCGACCTCGGCATCGCGCCGCTCATCCGCGCCGACGAGGCGGGcaccgcgcgcgcctccgccgactttggcaacctctccgtcgccggcgtcggggcgcctcggctcgccgccgccgccgccgtgctctacccgtcgcgccccgccgacatcgccgcgctgctgcgcgcgtcgtgcgcacgcccGGCGCCGTTCGCGGTGTCCGCGCGGGGGTGTGGCCACTCGGTGCACGGCCAGGCCTCCGCGCCCGACGGCGTCGTCGTCGACATGGCGTCGCTCGGCCGCCTGCAGGGCGGCGGCGCGCGGCGCCTCGCCGTGTCAGTGGAGGGGCGGTACGTCGACGCCGGCGGCGAGCAGCTGTGGGTGGACGTGCTGCGCGCGTCCATGGCGCACGGGCTCACGCCGGTGTCGTGGACAGACTACCTCCACCTCACCGTCGGCGGCACGCTGTCCAACGCCGGCATCAGCGGCCAGGCCTTCCGCCATGGCCCCCAGATTTCCAACGTGCTAGAGCTCGACGTCATCACCGGTGTCGGGGAGATGGTGACGTGCTCGAAggagaaggcgccggacctgttcgacgcggtgctgggcgggctggggcagttcggcgtcatcacgcgggcgcgcatcccgctcgcgccggcgccggcgagggcgcggtgggtgcggttcgtgtacacgacggcggcggcgatgacggccgaccaggagcgcctcatcgccgtcgatcgcgccggcggcgccggcgcggtgggcgggCTGATGGACTACGTCGAGGGCTCGGTCCACCTGAACCAGGGCCTGGTCGAGACCTGGCGCAcgcagccgcagccgccttcgccgtcctcctcctcctcctcatccttcttctccgacgccgacgaggcccgcgtcgccgcgctcgccaaggaggccggcggcgtgctgtatttcctcgagggcgccatctacttcggcggcgccgccgggccgtccgccgccgacGTTGACAAGAGGATGGATGTGCTGCGTCGCGAGCTGCGGCACGAGCGCGGGTTCGTGTTCGCGCAGGACGTGGCGTACGCCGGGTTCCTGGACCGCGTCCACGACGGCGAGCTCAAGCTCCGCGCCGCGGGGCTCTGGGACGTGCCGCACCCATGGCTGAACCTGTTCCTCCCCCGCTCCGGCGTCCTCGCCTTCGCCGACGGCGTCTTCCACGGCATCCTCAGCCGCACCCCCGCCATGGGCCCCGTCCTCATCTACCCCATGAACCGCAACAAGTGGGACAGTAACATGTCGGCAGTGATCACCGACGACGACGGTGACGAGGTGTTCTACACGGTGGGGATCCTGCGGTCGGCGGCGGCGGCCGGCGACGTGGGGAGGCTGGAGGAGCAGAACGACGAGATCTTGGGTTTCTGCGAGGTGGCCGGGATAGCCTACAAGCAGTACCTGCCTTACTACGGCAGCCAGGCAGAGTGGCAGAAGCGGCACTTCGGTGCCAATCTCTGGCCAAGATTCGTGCAGCGGAAGAGCAAGTATGATCCAAAGGCCATCCTGTCCCGTGGCCAGGGGATTTTCACGTCACCACTCGCATGA |
| **Synthetic gene sequence**  ATGAAGCAGGAGCAGGTGCGCATGGCAGTACTTCTTATGCTGAATTGTTTCGTGAAAGCGACCGCTCCGCCCCCTTGGCCGCCGTCCGCGAGCAGTGCGTCGTTCCTCGACGACCTCGGAGACCTCGGGATTGCACCCTTGATTAGGGCCGATGAGGCGGGGACCGCTAGAGCCAGCGCTGACTTCGGTAATCTGTCAGTGGCCGGCGTTGGGGCTCCTAGGTTGGCAGCGGCCGCCGCCGTGTTGTATCCAAGTAGACCCGCAGATATAGCCGCACTTTTGAGGGCAAGCTGCGCTAGGCCTGCGCCTTTTGCAGTGTCAGCAAGAGGCTGCGGCCATTCAGTGCATGGTCAGGCATCTGCACCGGATGGCGTTGTCGTCGACATGGCTTCTCTCGGTCGCCTGCAAGGCGGGGGTGCTAGGAGGTTGGCTGTGTCCGTCGAAGGCAGATACGTCGACGCTGGAGGTGAACAGCTGTGGGTTGATGTGCTTCGGGCGTCAATGGCTCACGGGCTCACACCCGTTTCGTGGACTGATTACCTGCACCTCACTGTGGGTGGCACCTTGTCGAATGCCGGTATCTCCGGACAGGCCTTCCGCCACGGTCCCCAGATTTCCAACGTACTTGAGCTGGACGTGATCACAGGGGTCGGCGAAATGGTGACGTGTTCCAAGGAGAAAGCGCCGGATCTCTTCGATGCGGTCCTTGGGGGCCTTGGCCAGTTTGGAGTCATTACGCGCGCGAGGATTCCCCTTGCGCCTGCCCCTGCCCGTGCGAGGTGGGTGAGATTTGTTTATACCACCGCAGCAGCTATGACTGCGGATCAAGAGCGTCTGATCGCTGTAGACCGCGCAGGAGGCGCCGGTGCGGTAGGCGGTCTGATGGATTACGTGGAAGGCTCAGTTCATCTGAACCAGGGGCTCGTAGAGACGTGGAGAACGCAACCGCAGCCACCATCCCCATCTTCATCGTCCTCTAGCTCTTTCTTCAGTGACGCCGACGAGGCGCGCGTTGCCGCTCTCGCCAAAGAGGCCGGCGGAGTCCTCTATTTTTTGGAGGGTGCGATCTATTTCGGTGGAGCCGCGGGACCGAGCGCGGCTGATGTTGACAAGCGTATGGATGTCCTCCGTCGCGAGTTGCGCCACGAGAGGGGATTCGTCTTTGCCCAGGACGTGGCCTACGCGGGCTTCCTCGATCGCGTCCACGATGGCGAACTTAAGCTGAGGGCCGCTGGGCTGTGGGACGTGCCACATCCGTGGTTGAATCTCTTTTTGCCCCGGTCCGGGGTGCTTGCGTTTGCAGATGGCGTCTTTCATGGGATATTGAGCCGGACCCCGGCCATGGGCCCTGTCCTGATCTACCCTATGAACCGGAATAAGTGGGATTCTAACATGTCCGCCGTCATCACAGACGACGACGGCGACGAGGTTTTCTATACAGTTGGGATCCTCCGGAGTGCCGCGGCTGCCGGCGATGTGGGCCGTCTCGAAGAACAAAACGACGAAATACTGGGCTTCTGCGAGGTTGCCGGCATAGCATACAAACAATACCTCCCATACTATGGAAGCCAGGCGGAGTGGCAGAAGCGGCACTTCGGGGCTAACCTGTGGCCACGCTTCGTGCAACGGAAGTCGAAGTACGATCCAAAGGCGATTCTTAGCCGGGGACAAGGAATCTTCACTTCGCCGCTGGCCTGA |
| **Protein sequence**  MKQEQVRMAVLLMLNCFVKATAPPPWPPSASSASFLDDLGDLGIAPLIRADEAGTARASADFGNLSVAGVGAPRLAAAAAVLYPSRPADIAALLRASCARPAPFAVSARGCGHSVHGQASAPDGVVVDMASLGRLQGGGARRLAVSVEGRYVDAGGEQLWVDVLRASMAHGLTPVSWTDYLHLTVGGTLSNAGISGQAFRHGPQISNVLELDVITGVGEMVTCSKEKAPDLFDAVLGGLGQFGVITRARIPLAPAPARARWVRFVYTTAAAMTADQERLIAVDRAGGAGAVGGLMDYVEGSVHLNQGLVETWRTQPQPPSPSSSSSSSFFSDADEARVAALAKEAGGVLYFLEGAIYFGGAAGPSAADVDKRMDVLRRELRHERGFVFAQDVAYAGFLDRVHDGELKLRAAGLWDVPHPWLNLFLPRSGVLAFADGVFHGILSRTPAMGPVLIYPMNRNKWDSNMSAVITDDDGDEVFYTVGILRSAAAAGDVGRLEEQNDEILGFCEVAGIAYKQYLPYYGSQAEWQKRHFGANLWPRFVQRKSKYDPKAILSRGQGIFTSPLA* |
