# Supplementary material for: Phenotypic, Transcriptomic, and Metabolomic Signatures of Root-Specifically Overexpressed OsCKX2 in Rice
Source: Front Plant Sci. 2021 Jan 20;11:575304. doi: 10.3389/fpls.2020.575304 (PMC7719687; doi:10.3389/fpls.2020.575304)
Supplement: Supplementary Table 2 — List of primers used in this study. [file Table_2.docx]

**Table S2.** List of primers used in this study.

| **Name** | **Forward primer (5'-3')** | **Reverse primer (5'-3')** |
| --- | --- | --- |
| *OsCKX2-syn* | GAGCAGTGCGTCGTTCCT | GCTTGCCCTCAAAAGTGC |
| *OsCKX2-en* | TTCGTCCGCCTCCTTCCT | CGACGCGCGCAGCAGCGC |
| *Actin* | GGAAGTACAGTGTCTGGATTGGAG | TCTTGGCTTAGCATTCTTGGGT |
| *OsPSK4* | GCCGTGCTGCTGATTTTC | GTGATGCTGCTGGGTGTAGA |
| *OsPUP6* | TCCCTGATGCAGCTCACGTT | TCGCCCTTCTTGTACCCGTC |
| *LBD11-1* | CCAGAACCAAGTCTCCCAGC | TCCACATGGACTCTTTCTTGAGG |
| *OsPsbR2* | GACCGAACCTGAAAGACGGT | CCAGCCAGCAGAATTCCAGA |
| *OsMST1* | TGACGTTCTCGGTGGTCATC | TAGACGCAGTACTCGTTCCC |
| *OsPME22* | ACTCGCTTCGCCAGTTCTAC | CTGGGGTGTATCAGGCTGTC |
| *OsPGL21* | AACTTGGCAGGGAGGTTCAG | CATGCAAAATGGGCAGGCTT |
| *OsAP25* | CCGAACTACACGTTCGGGTG | AGCGAGCCGGAGAAGTAGTA |
| *OsSub31* | GCTTACAGCGCCATGGAAAG | CCGGAGTAGTTCTTGCCGTT |
| *OsBGal1* | AGGAACCATCCGTCAACGAC | AGTCACAGTTGGATCGGCAG |
